# Diagnostic accuracy of single-, tri-, and four-phasic CT urography and radiation dose assessment: a systematic review and meta-analysis

**DOI:** 10.1186/s12880-026-02690-9

**Published:** 2026-08-18

**Authors:** Shivanath Shanbhag, Nitika C. Panakkal, Prakashini Koteshwara, Suresh Sukumar, Priya Pattath Sankaran, Visakh Thrivikraman Nair, Jyoti Garg

**Affiliations:** 1 Department of Medical Imaging Technology, Manipal College of Health Professions, Manipal Academy of Higher Education, Manipal, Karnataka India; 2 Radio-Diagnosis & Imaging, Department of Radio Diagnosis & Medical Imaging, Kasturba Medical College, Manipal Academy of Higher Education, Manipal, Karnataka India

**Keywords:** CT urography, Diagnostic accuracy, Radiation dose, Dose optimization, Multiphase CT, Urinary tract imaging

## Abstract

**Background:**

Computed tomography urography (CTU) is widely used for evaluating urinary tract disorders, but the optimal number of acquisition phases remains uncertain because additional phases increase radiation exposure. This study aimed to evaluate the diagnostic accuracy of single-, tri-, and four-phase CT urography and to synthesize available evidence on radiation dose characteristics and dose-reduction strategies.

**Methods:**

A systematic review and meta-analysis were conducted in accordance with PRISMA 2020 guidelines and registered in PROSPERO. PubMed, Scopus, Embase, Web of Science, and CINAHL were searched for studies published between January 2000 and July 2026. From 2,247 identified records, 38 studies (26 diagnostic accuracy and 12 radiation dose studies) met the inclusion criteria. Diagnostic accuracy was pooled using a bivariate random-effects model, with phase-wise subgroup analyses where feasible. Radiation dose metrics (CTDI_vol_ and dose–length product) were synthesized using random-effects models. Study quality was assessed using QUADAS-2 for diagnostic accuracy studies and the Joanna Briggs Institute checklist for radiation dose studies.

**Results:**

For urothelial carcinoma, pooled sensitivity and specificity were 0.88 and 0.93, respectively. Compared with single-phase CTU (specificity 0.87), specificity increased to 0.95 for both tri-phase and four-phase CTU, while sensitivity remained high across protocols. For renal cell carcinoma, pooled sensitivity and specificity were 0.90 and 0.97, respectively; however, phase-wise comparisons were not feasible because of the limited number of eligible studies. Tri-phase CTU demonstrated a pooled CTDI_vol_ of approximately 9 mGy, with reported values ranging from 5 to 16 mGy across studies. Low-dose strategies, including phase reduction, low tube voltage, and advanced reconstruction techniques, achieved radiation dose reductions of 20–72% while maintaining diagnostic performance.

**Conclusions:**

CT urography provides high diagnostic accuracy across urinary tract pathologies. For urothelial carcinoma, tri-phase CTU provides the most favorable balance between diagnostic performance and radiation exposure. In contrast, single-phase CTU may be appropriate for patients with microscopic haematuria and a low pre-test probability of urothelial malignancy, whereas four-phase CTU may be reserved for patients with a high pre-test probability of urothelial carcinoma or equivocal findings requiring further characterization. Indication-based protocol selection and dose-optimization strategies should be adopted to minimize radiation exposure while maintaining diagnostic quality.

**Study registration:**

The review was developed and recorded in accordance with the PRISMA guidelines and has been registered with PROSPERO (CRD420251163062).

**Supplementary Information:**

The online version contains supplementary material available at https://doi.org/10.1186/s12880-026-02690-9.

## Introduction

CT urography (CTU) has gradually become the main imaging test for examining the urinary tract. In clinical practice, it has largely replaced intravenous urography because it tends to show both anatomy and disease with greater accuracy [1–4]. CTU is routinely used for investigating hematuria and suspected urinary tract malignancy; however, the detection of urothelial lesions remains challenging. Small papillary tumors, flat lesions, carcinoma in situ, and subtle mural abnormalities may be difficult to identify, particularly when contrast timing or phase selection is suboptimal [5–7]. Diagnostic performance is therefore closely dependent on the acquisition phase, as different phases contribute variably to lesion conspicuity in the renal pelvis, ureters, and bladder. In addition, CTU examination may be prolonged, with patients often remaining on the table for up to twenty minutes, depending on the protocol complexity [8–10].

In 2008, the European Society for urogenital Radiology (ESUR) published guidelines for CTU, recommending the appropriate number and timing of acquisition phases according to the clinical indication while emphasizing radiation dose optimization. Despite these recommendations, considerable variation in CTU protocols continues to exist across institutions [11]. An international assessment of CTU protocols, covering centres in 20 countries, showed considerable variability. Most hospitals reported using either three or six-phase approaches, with the six-phase protocol adding delayed acquisitions (3–10 min) to the standard non-contrast, arterial, nephrographic, and excretory phases, with median dose–length product (DLP) values varying widely, ranging from approximately 1793 to 3618 mGy·cm [9]. Protocol variation also reflects differences in contrast administration strategies, with single-bolus protocols requiring separate acquisitions for each enhancement phase, whereas split-bolus and triple-bolus techniques combine multiple enhancement phases into fewer acquisitions to reduce the number of scans and radiation exposure [1, 12]. In a four-phase CTU protocol, an additional corticomedullary phase is acquired. Although primarily used for assessment of the renal parenchyma, this early post-contrast phase has also been reported to provide the highest sensitivity for bladder cancer detection [13] the excretory phase remains essential for detailed inspection of the urinary tract and helps in defining the nature of any detected abnormalities [14]. Although additional phases may improve diagnostic confidence, they inevitably increase radiation exposure, necessitating a careful balance between diagnostic benefit and radiation safety. Accordingly, international radiological protection guidelines, including ICRP and the European Commission recommendations, advocates the use of diagnostic reference levels (DRLs) to support optimization of CT protocols while maintaining diagnostic image quality [15, 16].

As CTU becomes more widely used in routine clinical care, concerns about cumulative radiation dose have become more prominent. Patients who undergo CTU repeatedly may accumulate a substantial effective dose over time, and this has been associated with an increased long-term risk of radiation-related malignancy [17–19]. The concern is particularly relevant for individuals requiring repeated imaging because of chronic urological conditions, who may be exposed to higher cumulative radiation doses over time [18, 20]. Dose-reduction strategies such as automated tube-current modulation, iterative reconstruction, dual-energy CT with virtual non-contrast imaging, and deep learning- based reconstruction have shown promise in lowering radiation dose without compromising image quality [21–24]. The diagnostic contribution of the individual CTU phases remains inconsistently reported. Importantly, prior studies and reviews have rarely examined diagnostic accuracy and radiation dose together across different phase-based CTU protocols. This represents a key knowledge gap, as uncertainty persists regarding the optimal balance between diagnostic performance and radiation exposure. Accordingly, the present systematic review and meta-analysis aimed to evaluate the diagnostic accuracy of single-, tri-, and four-phase CT urography for urothelial carcinoma, renal cell carcinoma, and benign or non-malignant conditions, while also synthesizing evidence on associated radiation dose characteristics and dose-optimization strategies.

## Methods

### Protocol registration

The PRISMA 2020 guidelines were followed in the design and reporting of this systematic review [25]. The protocol for the review was prospectively registered with the International Prospective Register of Systematic Reviews to ensure methodological transparency and rigor.

### Selection criteria

The review included studies that met specific criteria: human participants who underwent CTU for urinary tract evaluation. Eligible studies included only original research, such as prospective or observational studies, comparative trials, and dose-optimisation research. Radiation dose analysis requires metrics such as the Computed Tomography Dose Index–Volume (CTDI_vol_), DLP, and effective dose, along with dose reduction techniques. For diagnostic accuracy, studies need to provide quantitative performance indicators, such as sensitivity, specificity, and overall accuracy. Additionally, studies examining the impact of dose reduction on image quality were included.

Studies were excluded if they did not involve clinical CTU examinations, including those conducted on phantoms or animals, radiomics-focused investigations, or research primarily addressing diagnostic reference levels (DRLs). Pediatric studies were excluded because CTU indications, protocol design, contrast timing, and dose-optimization strategies differ substantially from adult practice, limiting methodological comparability. Additional exclusions encompassed case reports, conference abstracts, and reviews lacking original data. Studies utilizing dual-source CT systems were excluded due to fundamental differences in acquisition geometry and spectral imaging capabilities that could confound phase-wise diagnostic and dose comparisons. Studies employing split-bolus or triple-bolus CTU protocols were also excluded, as these techniques combine multiple enhancement phases into a single acquisition, precluding phase-specific assessment of diagnostic accuracy and radiation dose.

### Data sources

A literature search was carried out to identify studies from January 2000 onward. Five databases were checked: PubMed, Scopus, Embase, Web of Science, and CINAHL. Only peer-reviewed articles were included, so grey literature and unpublished work were not considered. The search aimed to find studies that reported radiation-dose reduction methods and diagnostic performance in CT urography.

### Search strategy

A structured search strategy incorporating free-text keywords was employed to identify relevant studies. Separate searches were conducted for diagnostic performance and radiation doses in CTU. Keywords were combined relevant Boolean operators (AND, OR), and the search string included the following:

1. Diagnostic performance.

(“Computed Tomography” OR “CT Scan” OR CT) AND (“Urography” OR CTU) AND (“Diagnostic Performance” OR “Diagnostic Accuracy”) AND (“RCC” OR “UTUC” OR “Bladder cancer” OR “Bladder tumor” OR “TCC” OR “Renal cancer” OR “Kidney cancer” OR “Renal carcinoma” OR “Kidney carcinoma”).

2) Radiation dose optimization.

(“Computed Tomography” OR “CT Scan” OR CT) AND (“Low kVP” OR “Low Tube Voltage” OR “70 kVp” OR “Radiation Dose Optimization” OR “Dose Reduction Technique” OR “Dose Optimization” OR “Low Voltage” OR “Low Dose Scan” OR “80 kVp” OR “90 kVp” OR “100 kVp” OR “Phase Reduction”) AND (“Urography”).

### Selection process

The studies were selected by two independent reviewers (SSH, NP) using Rayyan software (https://new.rayyan.ai) for the systematic screening process. In the first phase, the titles and abstracts were assessed on the basis of predefined inclusion and exclusion criteria. In the following phase, the full texts of studies that seemed potentially eligible were evaluated. Any disagreements that arose during either phase were resolved through discussion and, if necessary, with input from a third reviewer (PK). A PRISMA flow diagram was used to document the entire selection process, and the reasons for exclusions during the full-text review were recorded.

### Data extraction

The relevant data were independently extracted by two reviewers (JG, VT) using a standardized Excel template. Disagreements in data interpretation were resolved by discussion; if consensus could not be reached, a third reviewer (PS) was consulted. Five studies required author contact because of missing or unclear information. Corresponding authors were contacted by email; however, no response was received after two contact attempts. These studies were retained using the available published data only, and variables with insufficient information for quantitative synthesis were excluded from the respective analysis.

### Extracted variables

We collected key details from each study that met the criteria. This included the author names, publication year, country, study type, and basic patient information such as sample size, age range, and sex. We also noted CTU protocol characteristics, including the number of acquisition phases, tube voltage, and any other reported scanning parameters. For radiation dose studies, CTDI_vol_, DLP, or effective dose was extracted.

### Data extraction for meta-analysis

We extracted 2 × 2 diagnostic data to calculate pooled sensitivity and specificity. Where 2 × 2 data were not directly reported, they were derived from the available diagnostic performance metrics together with the reported numbers of disease-positive and disease-negative cases using standard formulas [26]. Studies were stratified by disease category: urothelial carcinoma, renal cell carcinoma, and benign or non–malignant conditions and sub-stratified by the respective number of acquisition phases when sufficient data were available. For the phase-wise subgroup analysis, studies were categorized as single-, tri-, or four-phase CTU according to the acquisition protocols reported in the original study, with each eligible phase combination from studies evaluating multiple protocols analyzed as a separate dataset. Detailed phase classifications and acquisition phase combinations for each included study are provided in Supplementary Table 1. Studies lacking sufficient data to reconstruct 2 × 2 contingency tables were excluded from the quantitative diagnostic accuracy meta-analysis. Radiation-dose metrics were pooled only where adequate and comparable data were available.

### Risk of bias

The quality of the included studies was evaluated to determine the reliability of their findings and to identify potential sources of bias. For studies reporting diagnostic accuracy, the QUADAS-2 tool [27], was applied to assess four domains: patient selection, conduct of the index test, choice of reference standard, and appropriateness of timing and patient flow. Studies addressing radiation doses were assessed using the Joanna Briggs Institute checklist for analytical cross-sectional studies [28]. This checklist considers factors such as how clearly the inclusion criteria were defined, whether the measurements were valid, how confounders were handled, and whether the statistical methods were appropriate. Two reviewers (SS and PK) completed the assessments independently, and any differences were settled through discussion.

### Certainty of evidence

The certainty of evidence for the pooled diagnostic accuracy outcomes was assessed using the Grading of Recommendations Assessment, Development and Evaluation (GRADE) approach. Summary of findings tables were generated to present the overall certainty of evidence and the reasons for any downgrading across the domains of risk of bias, indirectness, inconsistency, imprecision, and publication bias according to the GRADE framework [29].

### Statistical analysis

Statistical analyses were performed using R (version 4.5.2) for the diagnostic accuracy meta-analysis and Jamovi (version 2.6.26) for the radiation dose meta-analysis. Diagnostic accuracy was summarized using the 2 × 2 data extracted from each study. Sensitivity and specificity were estimated using a bivariate random-effects model implemented in the mada package using the Reitsma function, and Hierarchical summary receiver operating characteristic (HSROC) curves were generated with their 95% confidence intervals. Heterogeneity in diagnostic estimates was assessed using I² statistic. For studies that reported radiation metrics, the continuous measures (CTDI_vol_, DLP, and effective dose) were pooled as mean values using a random-effects model, provided that the reporting formats and units were sufficiently comparable across studies. Publication bias was assessed, where applicable, using the Deeks funnel plot for diagnostic-accuracy meta-analyses and the funnel plot for pooled radiation-dose outcomes. Sensitivity analyses were performed to assess the robustness of the pooled estimates by restricting the analysis to prospective studies and studies with a low risk of bias, and by conducting leave-one-out analyses where applicable.

## Results

### Study selection

**Fig. 1:**
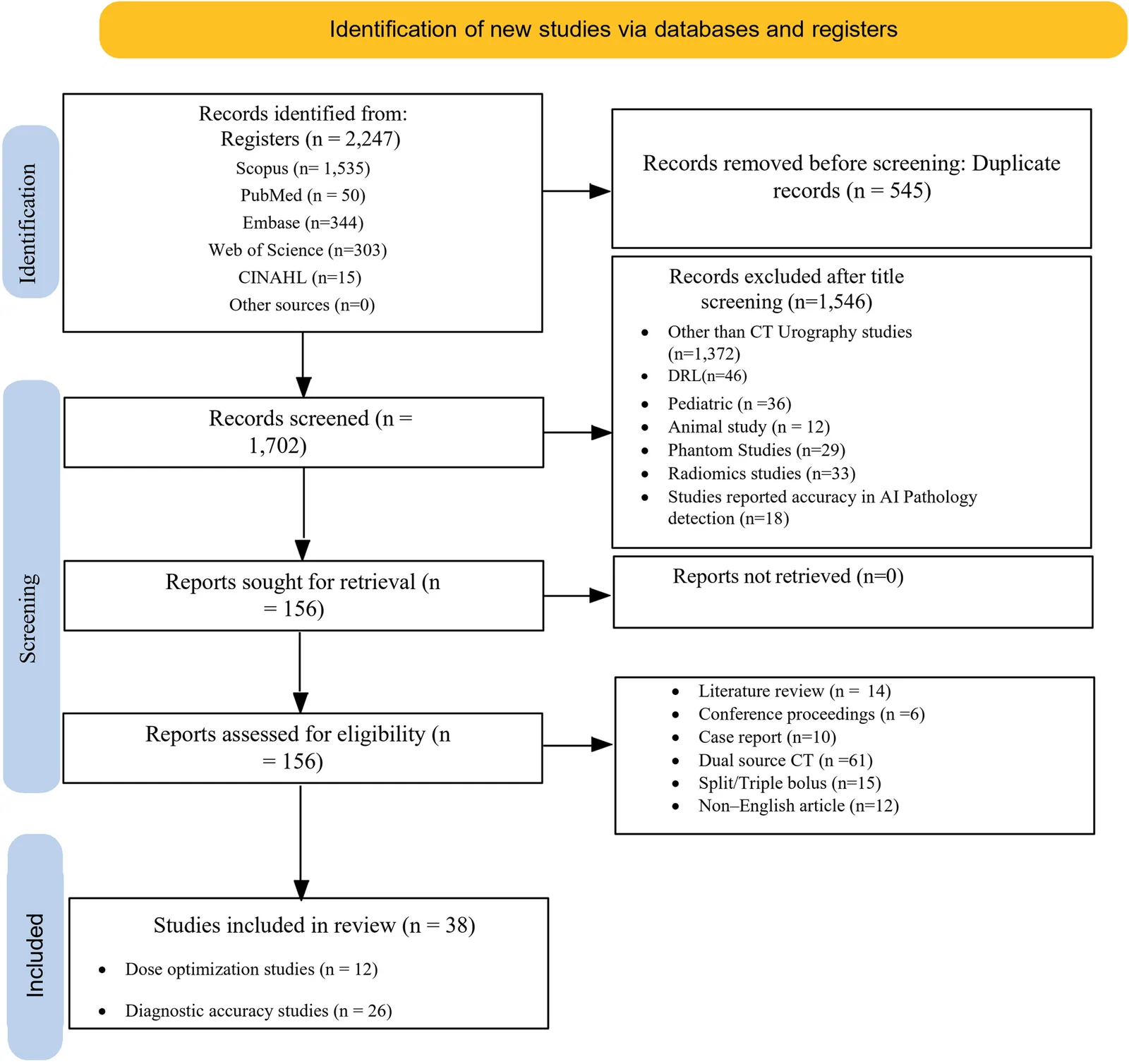
PRISMA 2020 flow diagram summarizing study selection for the systematic review and meta-analysis

A systematic search of five electronic databases identified 2,247 records. Following duplicate removal, screening, and full-text eligibility assessment, 38 studies met the inclusion criteria and were included in the systematic review, comprising 26 diagnostic accuracy studies and 12 radiation dose optimization studies (Fig. 1).

### Study characteristics

A total of 38 studies met the inclusion criteria, comprising 26 diagnostic performance studies (66%) and 12 radiation dose optimization studies (34%) published between 2006 and 2024 (Tables 1 and 2). Sample size ranged from 20 to 1,692 participants.

**Table 1:** Summary of study characteristics and diagnostic performance metrics of included CT urography studies

Author (Year)	Indication	Study design	Sample Size & Age	Contrast media	kVp/mAs Modulation	Phases	Sensitivity	Specificity	TP	FP	FN	TN	Reference Standard
Turney et al. 2006 [30]	Bladder tumours	Prospective Study	200 (> 40 Years)	100 mL (Iopamidol 300)	120 kVp, 170–220 mAs	UE, CM1,CM2	0.93	0.99	42	1	3	115	Flexible Cystoscopy and Histopathology
Park et al. 2007 [31]	Bladder lesion	Prospective Study	118 (62 ± 14)	Iopromide 300 or Iopamidol 300	120 kVp, 130–150 mAs	UE, CMP, PP, EP	95%	91%	60	4	3	51	Conventional cystoscopy and Histopathology
	Bladder lesion						95%	93%	60	5	3	50	
Wang et al.2008 [32]	Transitional Cell Carcinoma	Prospective Study	139 (61.12 ± 19.55)	120 ml Omnipaque 350	120 kVp, 30–127 mAs	UE, AP, NP, EP	0.961	0.988	74	6	3	483	Histological evidence
	Transitional Cell Carcinoma						0.857	0.98	66	10	11	479	
Sadow et al. 2008 [33]	Bladder cancers	Retrospective Study	779 (18–92)	100 mL of Iopromide (Ultravist 300)	120 kVp, 155–280 mAs	UE, NP, EP	78.5%	94.2%	117	40	32	649	Pathological Findings
Hwang et al. 2010 [34]	Bladder tumor	Retrospective Study	93 (63.38 ± 12.13)	125 to 140 ml of Iopromide (Ultravist 300)	120 kVp, 170–280 mAs	UE, NP, EP	86.70%	96.80%	26	2	4	61	Cystoscopy biopsy
	Urothelial cancer		50 (63.38 ± 12.13)				60.00%	80.00%	15	5	10	20	
Jinzaki et al. 2010 [35]	UTUC	Retrospective Study	104 (40–88)	Iohexol (Omnipaque 300)	NR, 110–380 mAs	UE, NP, EP	93.50%	94.80%	43	3	3	55	Histopathologic examination
							80.4%	81%	37	11	9	47	
Wang et al.2010 [36]	UUTTCC	Retrospective Study	60 (60.73 ± 12.95)	50 ml Omnipaque 350	NR	UE, AP, NP, EP	0.958	1	23	0	1	215	Histological confirmation
Kupershmidt et al. 2011 [37]	Upper Urinary Tract Tumors	Retrospective Study	20 (44–83)	Iohexol, (Omnipaque 300)	120 kVp, 230–330 mAs	PV	100%	97.5%	20	1	0	39	Histopathology, Biopsy
							100%	95%	20	2	0	38	
Metser et al. 2012 [38]	Urothelial malignancy-Upper tract tumor	Prospective Study	77 (28–88)	100–130 mL of Iodixanol (Visipaque 320)	120 kV, ATMC	UP, EP	88.2%	90.50%	30	12	4	114	Histopathologic findings
	Bladder tumors					UP, EP	92.60%	83.3%	59	16	5	80	
	Upper tract tumor					UP	85%	NR	29	5	5	121	
	Upper tract tumor					EP	79%	NR	27	10	7	116	
	Bladder tumors					UP	90.6%	88.80%	58	10	6	86	
	Bladder tumors					EP	73.4%	91.7%	47	8	17	88	
Kim et al. 2013 [39]	Bladder Cancer	Prospective Study	121 (46–88)	200 mL of iopromide (Ultravist 300)	120 kVp, 10–510 mAs	NP	90.3%	NR	56	18	6	200	Histologic findings
	Bladder Cancer					Pyelographic phase	72.6%	NR	45	44	17	174	
	Upper tract lesion					NP	90.9%	NR	20	48	2	210	
	Upper tract lesion					Pyelographic phase	72.7%	NR	16	87	6	171	
	Bladder Cancer					NP, Pyelographic phase	91.9%	82.60%	57	38	5	180	
	Upper tract lesion					NP, Pyelographic phase	81.8%	90.70%	18	24	4	234	
Kwon et al. 2014 [40]	Urolithiasis	Prospective Study	116 (48.9 ± 16.2)	NR	100 kVp, 60 mAs	UE	87.80%	97.00%	NR	NR	NR	NR	NR
	Urolithiasis						92.40%	95.20%					
Capalbo et al. 2015 [41]	Bladder cancer	Prospective Study	169 (71.4 ± 18.7)	Ioversol, 350 mgI/mL	120 kVp, Dose modulation	UE, AP, PP, NP, EP	96.30%	86.40%	106	4	8	51	Cystoscopy and histological examination
Helenius et al. 2015 [42]	Bladder tumours	Observational cohort study	435 (63 ± 16)	Iohexol 350 mgI/ml (Omnipaque)	120 kVp, 60–80 mAs	UE, CMP, NP, EP	0.87	0.99	48	5	7	375	Flexible cystoscopy
Recaldini et al., 0.2015 [43]	UUTTCC	Retrospective	66 (41–89)	Iopamiro 300	120 kVp, 10–500 mAs	UE, NP, EP	90.50%	84.40%	19	7	2	38	Histopathologic findings
Taniguchi et al. 2016 [44]	Acute pyelonephritis	Retrospective	100 (19–70)	NR	120 kVp, 240 mAs	UE, NP, EP	84%	95%	50	2	9	39	NR
	Acute pyelonephritis					UE, NP, EP	94%	94%	55	2	4	39	
	Acute pyelonephritis					NP	80%	96%	47	2	12	39	
	Acute pyelonephritis					NP	84%	95%	50	2	9	39	
	Urolithiasis					UE, NP, EP	87%	98%	10	2	1	87	
	Urolithiasis					UE, NP, EP	93%	99%	10	1	1	88	
	Urolithiasis					NP	80%	98%	9	2	2	87	
	Urolithiasis					NP	88%	100%	10	0	1	89	
	Renal abscesses					NP	80%	98%	10	2	2	86	
	Renal abscesses					NP	60%	100%	7	0	5	88	
Grahn et al. 2016 [45]	UTUC	Prospective study	148 71(mean)	Ultravist 370/Iomeron 400	NR	UE, AP, NP, EP	0.89	0.51	55	20	7	21	Cytopathological and histopathological
Trinh et al. 2017 [46]	Bladder cancers	Retrospective	95 (18–95)	75–100 ml of Iohexol 350 (Omnipaque)	120 kVp, 155–280 mAs	UE, NP, EP	86.30%	92.40%	32	39	5	486	Cystoscopy
	Urothelial cancer		148 (18–95)				0.86	0.91	50	8	8	82	
Kravchick et al. 2017 [47]	UTUC	Retrospective	140 (69.7 ± 16.98)	Iopamidol 300	NR	UE, NP, EP	66.7%,	98.50%	6	2	3	129	Ureteroscopy and cystoscopy
Tan et al. 2018 [48]	UTUC	Prospective multicenter observational study	1,692 (57–76)	NR	NR	NR	NR	99.6	NR	NR	NR	NR	Histopathological examination
	Renal cancer						NR	99.9	NR	NR	NR	NR	
	Bladder cancers						80.5	97	NR	NR	NR	NR	
Nazir et al.2019 [49]	Renal carcinoma	Cross-sectional study	100 (53.74 ± 11.39)	100mL Iopromide	120 kVp, 155–280mAs	UE, NP, EP	70.20%	81.40%	40	8	17	35	Histopathological examination
Rud et al. 2019 [50]	Upper urothelial cell carcinomas	Retrospective	376 (18–96)	Iohexol 350 mg/mL (Omnipaque)	NR	UE, CMP, NP, EP	100%	99%	7	1	0	368	Biopsy results
	RCC						100%	100%	4	1	0	371	
David et al. 2020 [51]	UUT Cancer	Retrospective	580 (21–92)	Omnipaque 300	NR	UE, NP, EP	100%	99.1	12	5	0	558	Histological confirmation
	RCC						100%	100%	8	0	0	567	
	UTUC						100%	99.10%	4	5	0	566	
Han et al. 2020 [52]	Bladder lesion	Retrospective	192 (60 ± 14)	Iopamidol 300 mg	120 kVp, 130–150 mAs	UE, BS, NP, EP	94%	96%	92	4	6	90	Conventional cystoscopy
Toda et al. 2021 [53]	RCC	Retrospective multicenter study	453 (62.0 ± 12.1)	NR	80–140 kV	NP	84.3	92.3	382	NR	71	NR	Histological confirmation
	RCC		132 (59.3 ± 11.0)		120 kV	NP	84.8	90.2	112	13	20	119	
Galtung et al. 2023 [54]	Urothelial carcinoma	Prospective study	308 (18–96)	Iohexol 350 mg/ml (Omnipaque)	NR	UE, CMP, NP, EP	93.3	83.70	40	28	2	192	Histologically findings
	Urothelial carcinoma					UE, CMP, NP, EP	82.5	95.4	33	11	4	229	
	Urothelial carcinoma					NP	91.3	90.3	42	23	4	215	
	Urothelial carcinoma					NP	90.2	93.9	37	15	4	229	
	Bladder UC					UE, CMP, NP, EP	92.3	89	36	25	3	202	
	Bladder UC					UE, CMP, NP, EP	87.9	96.4	29	9	4	240	
	Bladder UC					NP	95	90.7	38	22	2	214	
	Bladder UC					NP	89.2	95.2	33	12	4	240	
Galtung et al. 2024 [55]	RCC	Prospective study	308 (53–77)	Iohexol 350 mg/ mL (Omnipaque)	NR	UE, CMP, NP, EP	100%	97%	7	9	0	290	Histologically findings
	RCC					UE, CMP, NP, EP	100%	99%	7	3	0	295	
	RCC					NP	100%	98%	7	9	0	292	
	RCC					NP	86%	99%	6	2	1	295	

UE – Unenhanced, CMP - Corticomedullary phase, PP - Portal Venous Phase, AP - Arterial Phase, NP - Nephrographic Phase, EP - Excretory Phase, NR – Not reported

**Table 2:** Summary of study characteristics and radiation dose parameters of CT urography dose-optimization studies

Author (Year)	Country	Study Design	Indication	Sample Size	Age	Scanner	Phases	Slice Thickness	kVp	mAs / Modulation	Pitch	Recon Method	CTDIvol (mGy)	DLP (mGy·cm)	ED (mSv)
Meindl et al. (2007) [56]	Germany	Retrospective	Flank pain, genitourinary tumor suspicion or confirmation, retroperitoneal or pelvic inflammatory/neoplastic disease	45	Females: 60 ± 11.55; Males: 56 ± 17.62	4-row MDCT scanner (MX 8000, Philips Medical Systems)	PP/NP, EP1,EP2	3.2 mm	120 kVp	30 mAs for patients < 80 kg; 50 mAs for ≥ 80 kg	0.875	Standard reconstruction techniques	2.1 mGy (< 80 kg patient) and 3.4 mGy (≥ 80 kg patient)	NR	Average effective dose: 1.1 mSv for males and 1.8 mSv for females
Dahlman et al. (2011) [10]	Sweden	CS	Macroscopic hematuria Urinary tract infection renal abnormalities advanced stone disease	27	74 ± 9	Siemens Sensation 16 (16-MDCT scanner with Akron x-ray tube)	UE, CP, EP	Axial: 5/5 mm and 1/1 mm; Coronal: 5/5 mm	120	Normal-dose unenhanced: 100; Normal-dose corticomedullary: 120; Normal-dose excretory: 100; Low-dose unenhanced: 20; Low-dose excretory: 40	1	Axial and coronal images	Unenhanced phase: Normal-dose 7.0 mGy, Low-dose 1.5 mGy; Corticomedullary phase: 9.4 mGy; Excretory phase: Normal-dose 6.9 mGy, Low-dose 2.7 mGy	NR	Unenhanced phase: Normal-dose: 4.9 mSv; Low-dose 1mSv; Corticomedullary phase:6.5mSv; Excretory phase: Normal-dose 4.8mSv, Low-dose 1.9 mSv
Lee et al. (2012) [57]	Republic of Korea	Retrospective comparative study	Evaluation of flank pain and hematuria	25	100 kVp: 26.7 ± 2.6, 120 kVp: 25.5 ± 3.4	128-slice MDCT scanner (Definition AS+, Siemens Healthcare)	UE, NP, EP	1 mm	100 kVp and 120 kVp	180 mAs	1	soft convolution kernel (B30f)	100 kVp: precontrast - 5.01 ± 0.92, nephrographic - 8.24 ± 1.5, excretory phase - 5.00 ± 0.92; 120kVp: precontrast - 8.9 ± 1.2, nephrographic − 8.6 ± 1.17, excretory phase - 8.8 ± 1.25	100kVp: 867.6 ± 210.8, 120kVp: 1272.4 ± 218.7	100kVp: 13.0 ± 3.1 mSv, 120kVp: 19.1 ± 3.3 mSv
Juri et al. (2013) [58]	Japan	Prospective	Macroscopic hematuria	30	66.2	Aquilion ONE, Toshiba Medical Systems Corp	UE, NP, EP	Nephrographic phase: 3.0 mm; excretory phase: 1.0 mm	120 kVp	10–550 mA	0.53	Routine-dose CT: Filtered Back Projection (FBP); Low-dose CT: Adaptive Iterative Dose Reduction 3D (AIDR 3D)	unenhanced: 3.8mGy; Nephrographic phase: Routine-dose 14.5 mGy, Low-dose 4.2 mGy; Excretory phase: Routine-dose 9.2 mGy, Low-dose 2.7 mGy	NR	NR
Juri et al. (2013) [59]	Japan	Prospective study	Macroscopic hematuria	30	66.1	Toshiba 320-MDCT scanner (Aquilion ONE)	UE, NP, EP	Axial images: 1.0 mm; coronal MPR: 3 mm	120 kV	10–550 mA	0.53	Routine-dose images: Filtered Back Projection (FBP); Low-dose images: Adaptive Iterative Dose Reduction 3D (AIDR 3D)	Routine-dose mean CTDIvol: 8.17 mGy; Low-dose mean CTDIvol: 4.42 mGy	Routine-dose mean DLP: 379.74 mGy·cm; Low-dose mean DLP: 206.10 mGy·cm	Routine-dose mean ED: 5.7 mSv; Low-dose mean ED:3.1 mSv
Al-Amin et al. (2015) [60]	Bulgaria	Prospective patient study	Asymptomatic Haematuria renal colic voiding dysfunction palpable mass	91	56.4	Aquilion 64 (Toshiba)	UE, CP, NP, EP	5 mm	120 / 100 / 80	120 kV: 219 mAs; 100 kV: 163 mAs; 80 kV: 114 mAs	NR	Axial reconstructions + 3D reformats	Unenhanced:120 kV: 38.8,100 kV: 29.8, 80 kV: 13.6 CTU: 120 kV: 38.0, 100 kV: 20.0, 80 kV: 13.8	Unenhanced:120: 1733.4, 100 kV: 825.4, 80 kV:711.8 CTU: 120 kV:1476.5, 100 kV: 694.0, 80 kV:536.9	120 kV-22.5 mSv; 100 kV: 7.8mSv; 80 kV-5.2 mSv
Hwang et al. (2015) [61]	Republic of Korea	Prospective	Evaluation for hematuria, urinary abnormalities, pain, dysuria, lower urinary tract symptoms, follow-up urinary stone disease	63	57.3 ± 8.3	64-channel multidetector CT scanner	UE, CP, EP	5 mm axial slice thickness, 3 mm coronal and sagittal	Low-concentration protocol: 80 kVp; Conventional protocol: 120 kVp	Low-concentration protocol: 330 mAs; Conventional protocol: 120 mAs	0.891	Filtered back projection (FBP) for both groups	Low-concentration:- Precontract: 4.44 ± 0.69, Corticomedullary: 5.97 ± 2.09, Excretory: 4.62 ± 1.85; Conventional protocol:- Precontrast: 4.64 ± 1.05, Corticomedullary: 6.58 ± 2.78, Excretory: 7.62 ± 2.20	80 kVp: Low-concentration:- Precontrast: 227.9 ± 46.5, Corticomedullary: 266.0 ± 107.0, Excretory: 237.5 ± 98.4, CTU: 735.6 ± 217	Low Concentration 8o kVp: 10.17 ± 2.66 mSv; Conventional protocol 120 kVp: 12.79 ± 3.94 mSv
Zhou et al. (2016) [62]	China	Retrospective	NR	45	60.0 ± 1.3	Discovery CT 750 HD (GE Healthcare)	UE, NP, EP	5 mm	Group A: 80 or 100; Group B: 120	NR	0.984	Group A: 50% ASiR; Group B: Standard FBP	Group A: 9.26 ± 2.77; Group B: 16.19 ± 5.60	Group A: 368.86 ± 119.38; Group B: 674.38 ± 239.37	Group A:5.53 ± 1.79 mSv; Group B: 10.12 ± 3.59 mSv
Kim et al. (2018) [63]	Republic of Korea	Prospective, multi-institutional, randomized controlled trial	Evaluation of urinary tract symptoms	299	50 ± 12.8	Multi-detector CT scanners (at least 64 channels) from multiple vendors	UE, CP, EP	5 mm axial, 3 mm coronal/sagittal planes	LVLC-CTU group: 80 kVp (low tube voltage); Conventional CTU group: 120 kVp	Automatic tube current modulation	0.65 or 0.891 depending on scanner	Filtered Back Projection (FBP) and various Iterative Reconstruction (IR) algorithms specific to each vendor (iDose4, ASIR, SAFIRE)	LVLC-CTU group: 9.95 ± 9.79 mGy; Conventional: 12.88 ± 8.34 mGy	LVLC-CTU: 381.71 ± 269.06; Conventional: 562.16 ± 292.06	LVLC-CTU: 5.73 ± 4.04 mSv; Conventional: 8.43 ± 4.38 mSv
Garba et al. (2019) [64]	Nigeria	Prospective	Evaluation of urinary tract abnormalities	56	40.6 ± 16.0	Toshiba Aquillion-prime 160-slice MDCT scanner	UE, AP, EV Phase, LV phase, EP	5 mm	116.07 ± 8.02	147.09 ± 46.82	0.65	NR	9.67 ± 4.42	2320 ± 1132	35.04 ± 16.80 mSv
Cheng Y et al. (2021) [65]	China	Prospective	CT urography for urinary tract lithiasis, tumor, hematuria	52	LD-CTU: 57.2 ± 11.2, C-CTU: 60.3 ± 11.1	Revolution CT, GE Healthcare	UE, NP, EP	1.25 mm	100 kV	Auto mA	0.992	LD: DLIR; C-CTU: ASIR-V 50%	LD: 2.95 ± 0.44; C: 9.6 ± 1.67	LD: 134.29 ± 29.45; C: 460.59 ± 97.93	LD:2.01 ± 0.44 mSv; C: 6.9 ± 1.46
Saidu et al. (2024) [66]	Nigeria	Prospective comparative study	Patients referred for CT urography	32	Group A: 39.6 ± 12, Group B: 40.4 ± 17	Toshiba Aquilion Prime 160-slice	UE, NP, EP/SP	5 mm	100 (standard), 80 (optimised)	100 kV: 355 mAs, 80 kV: 440 mAs	0.813	Standard reconstruction algorithm (IR)	100 kV: 6.98 ± 2.22; 80 kV: 5.16 ± 1.29	100 kV: 952.29 ± 302.82; 80 kV: 685.92 ± 188.58	NR

NR – Not Reported, CS – Cross-sectional

Most diagnostic performance studies used single-, tri-, or four-phase CT urography protocols and have focused on urothelial carcinoma [34, 38, 46, 54], bladder tumors [30, 31, 33, 39, 67], renal cancer [48–51, 53, 55], urolithiasis [40, 44], and pyelonephritis [44]. The majority of studies employed slice thicknesses between 1 and 5 mm, variable or automatic mAs modulation, and 120 kVp. Histopathology, ureteroscopy, or cystoscopy served as the reference standards, with reported sensitivity ranged from 65% to 100%, whereas the specificity ranged from 80% to 99%.

Radiation dose optimization strategies included reduced tube voltage (80–100 kVp) [57, 60–63, 65, 66], automatic exposure controls [63, 65], iterative reconstruction techniques [58], and phase reduction, are some of the techniques used in radiation dose optimization research. Compared with standard protocols, reported CTDI_vol_ values ranged from 2.9 to 16.2 mGy, corresponding to a radiation dose reduction of 20–72%.

### Participant characteristics

The study populations varied widely, with sample sizes from 20 to over 1,600 participants aged 20 to 88 years. Most studies had a larger number of male patients due to the higher incidence of urinary tract disease in men. The mean participant age was typically between 40 and 75 years, with many papers reporting broad age ranges. CT urography was performed for clinical reasons like hematuria, suspected cancer, renal tumors, kidney stones, and urinary tract infections. No studies included children or pregnant patients, and most required adequate renal function and the ability to undergo multiphase contrast-enhanced CT for accurate comparisons across protocols.

### Variation in reference standards across studies

Across the included studies, single-phase CTU, particularly those assessing stones or benign inflammatory conditions, was generally validated against clinical or imaging follow-up, such as documented stone passage or symptom resolution. In contrast, multiphase CTU studies evaluating urothelial carcinoma or renal masses predominantly used cystoscopy, ureteroscopy, biopsy, or surgical histopathology as the reference standard. This distinction should be considered when comparing diagnostic performance across phase groups.

### Comparison of diagnostic performance across CTU phases and urinary tract locations

#### Meta-analysis

A meta-analysis was conducted to evaluate the diagnostic performance of CTU for Urothelial carcinoma (UC), renal cell carcinoma (RCC). A phase-wise comparison of CTU protocols was also conducted to investigate the impact of acquisition strategy on diagnostic accuracy. In addition, radiation dose parameters, CTDI_vol_, and DLP were meta-analyzed. In contrast, effective dose (ED) and data for benign/non-malignant conditions were synthesized descriptively because of the limited number of eligible studies. The results are presented in the following sections.

### Test quality for the detection of urothelial carcinoma

**Fig. 2:**
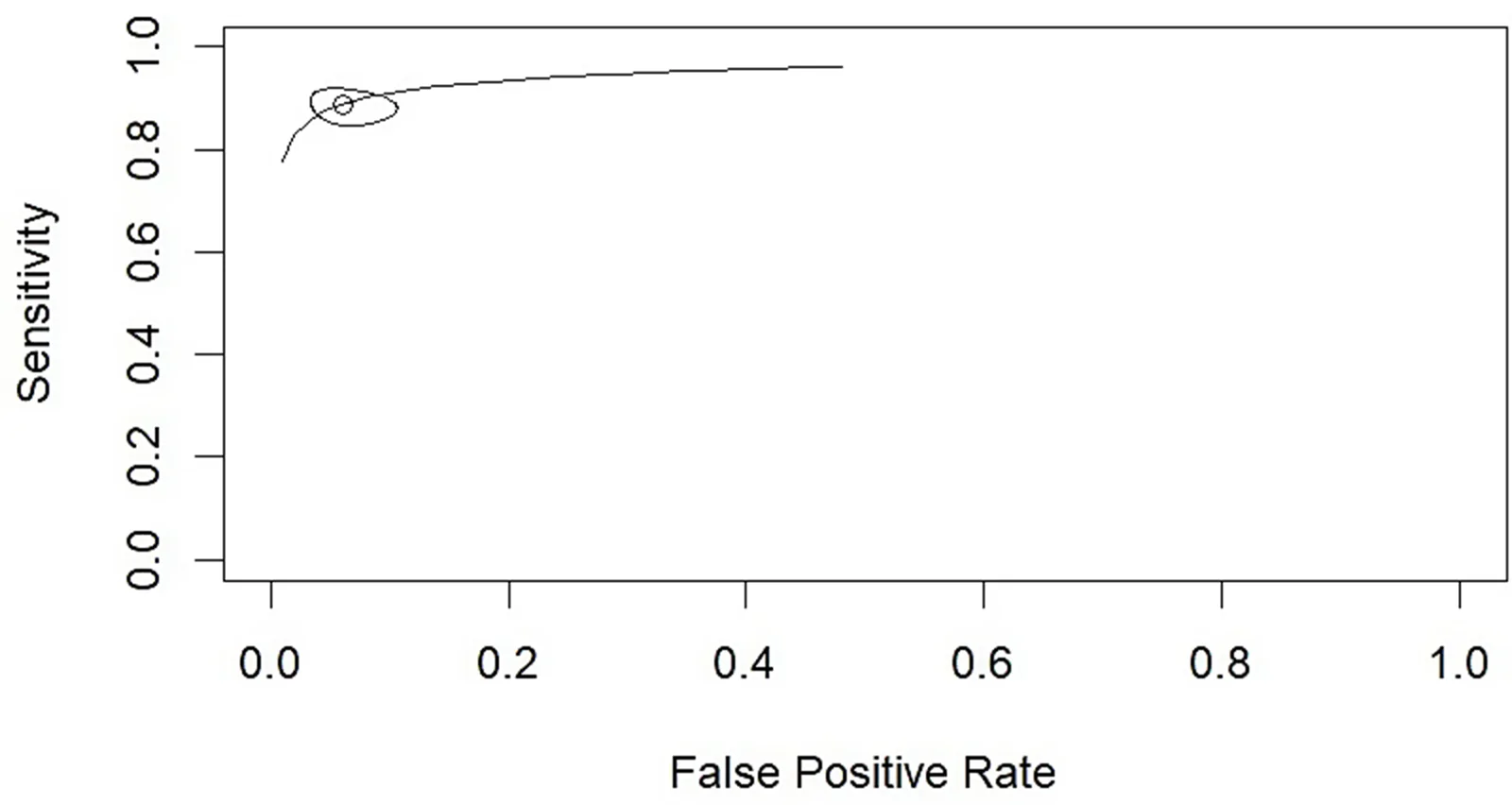
HSROC curve for the detection of urothelial carcinoma based on 20 studies (*n* = 4418 patients). The pooled AUC was 0.94, with low heterogeneity (I^2^ = 11.9%). The open circle represents the pooled summary estimate, the solid line the HSROC curve, and the ellipse the 95% confidence region

**Fig. 3:**
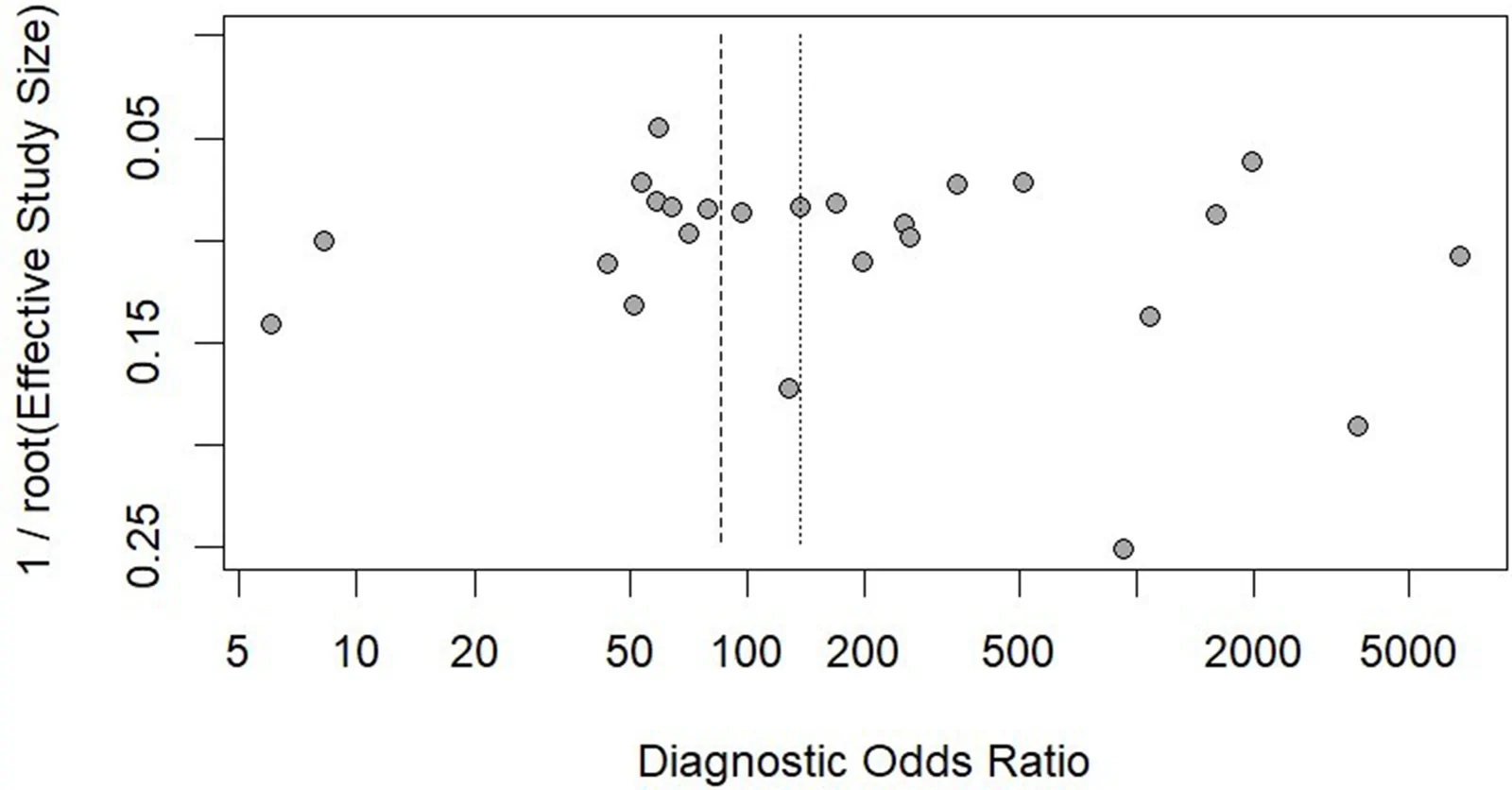
Deeks’ funnel plot for assessment of publication bias in studies evaluating the diagnostic accuracy of CT urography for urothelial carcinoma

A bivariate random-effect model was applied to pool diagnostic performance measures across included studies. The pooled sensitivity was 0.88 (95% CI: 0.85–0.91), and the pooled specificity was 0.93 (95% CI: ~ 0.90–0.96). The HSROC curve (Fig. 2) demonstrated excellent overall discrimination, with the summary point located in the high-sensitivity and low-false-positive-rate region. The AUC was 0.94, indicating strong discriminatory ability. The confidence region was compact, and heterogeneity was minimal (I^2^ = 11.9%), reflecting consistent diagnostic performance across studies. Deeks funnel plot asymmetry test demonstrated no significant evidence of publication bias (t = 0.34, *p* = 0.739), suggesting a low likelihood of small-study effects (Fig. 3).

### Sensitivity analysis

**Fig. 4:**
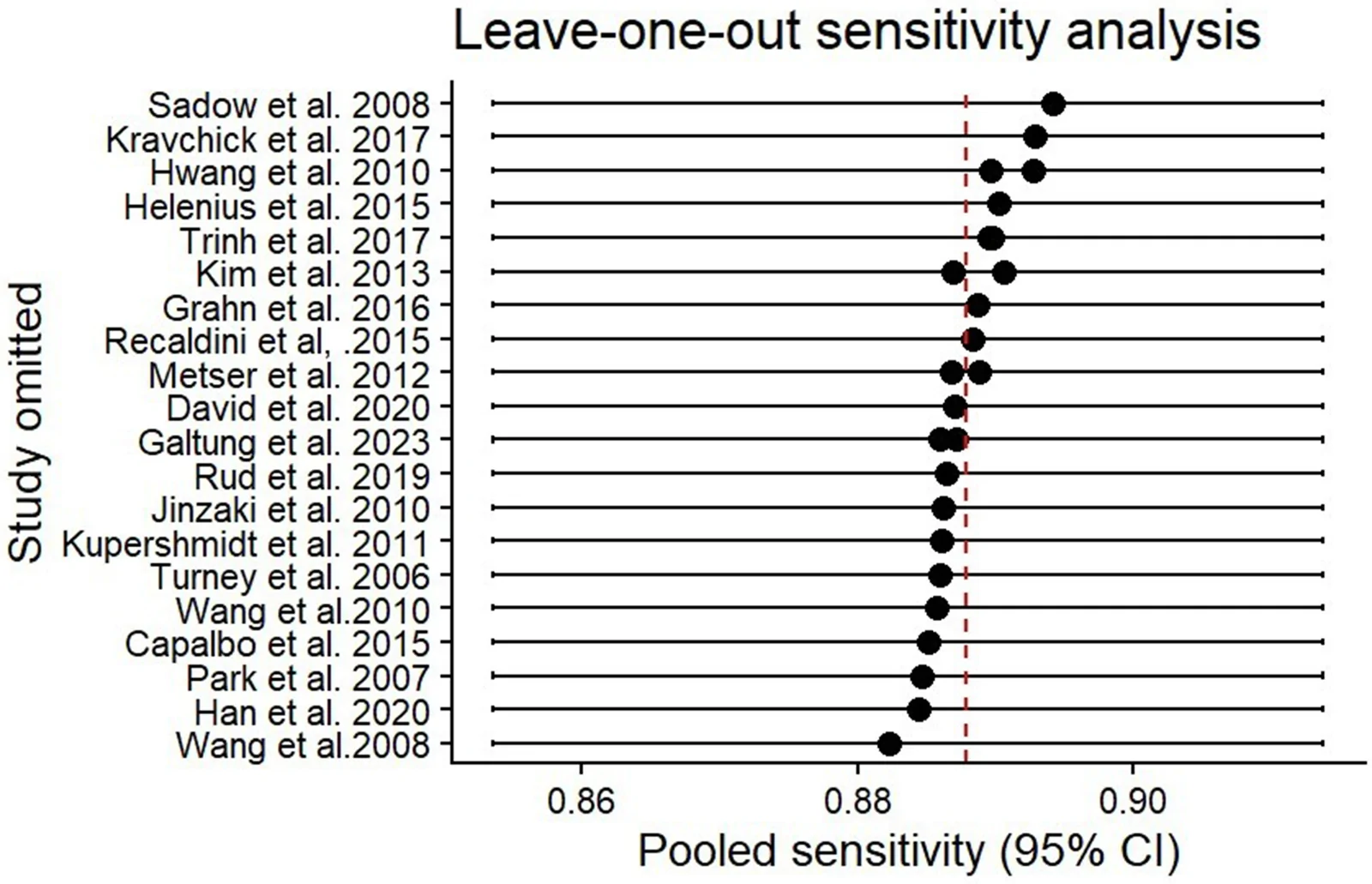
Leave-one-out sensitivity analysis for the urothelial carcinoma diagnostic accuracy meta-analysis. Each point represents the pooled sensitivity obtained after sequentially excluding one study from the meta-analysis. The red dashed vertical line indicates the original pooled sensitivity estimate

Sensitivity analysis demonstrated the robustness of the pooled diagnostic accuracy estimates. Restricting the meta-analysis to prospective studies yielded a pooled sensitivity of 0.92 (95% CI: 0.89–0.94), a pooled specificity of 0.90 (95% CI: 0.82–0.95). and an AUC of 0.93, with low heterogeneity (I^2^ = 0%). Restricting the analysis to studies with a low risk of bias yielded comparable results: a pooled sensitivity of 0.90 (95% CI: 0.86–0.92), a pooled specificity of 0.94 (95% CI: 0.90–0.96), and an AUC of 0.95 (I^2^ = 19.9%). Leave-one-out sensitivity analysis demonstrated that sequential exclusion of individual studies resulted in only minimal variation in pooled sensitivity (0.882–0.894) relative to the original pooled estimate (0.888), confirming that the overall findings were stable and were not unduly influenced by any single study (Fig. 4).

### Test quality for the detection of renal cell carcinoma

**Fig. 5:**
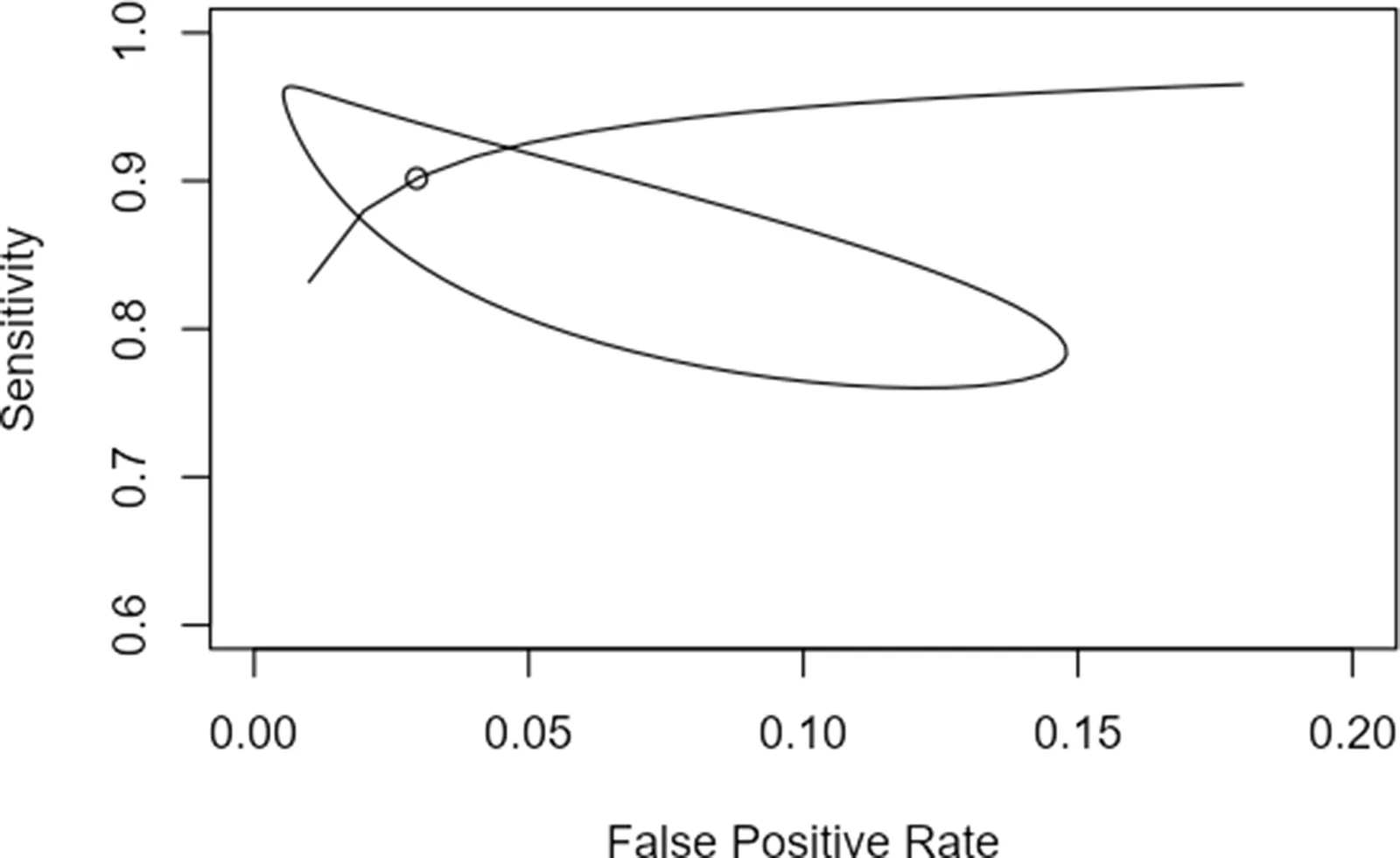
HSROC curve for the detection of renal cell carcinoma based on 5 studies (*n* = 1496 patients). The pooled AUC was 0.968, with negligible heterogeneity (I^2^ = 0%). The open circle represents the pooled summary estimate, the solid line the HSROC curve, and the ellipse the 95% confidence region

A bivariate random-effects model was applied to pool diagnostic performance measures for CT in the detection of renal cell carcinoma. The pooled sensitivity was 0.90 (95% CI: 0.79–0.95), and the pooled specificity was 0.97 (95% CI: 0.89–0.99). The HSROC curve (Fig. 5) showed the summary operating point in a region corresponding to high sensitivity and a very low false-positive rate, reflecting excellent diagnostic accuracy. The model yielded an AUC of 0.968. The confidence region was compact, and between-study heterogeneity was negligible (I^2^ =0%), indicating highly consistent diagnostic performance across studies.

### Sensitivity analysis

**Fig. 6:**
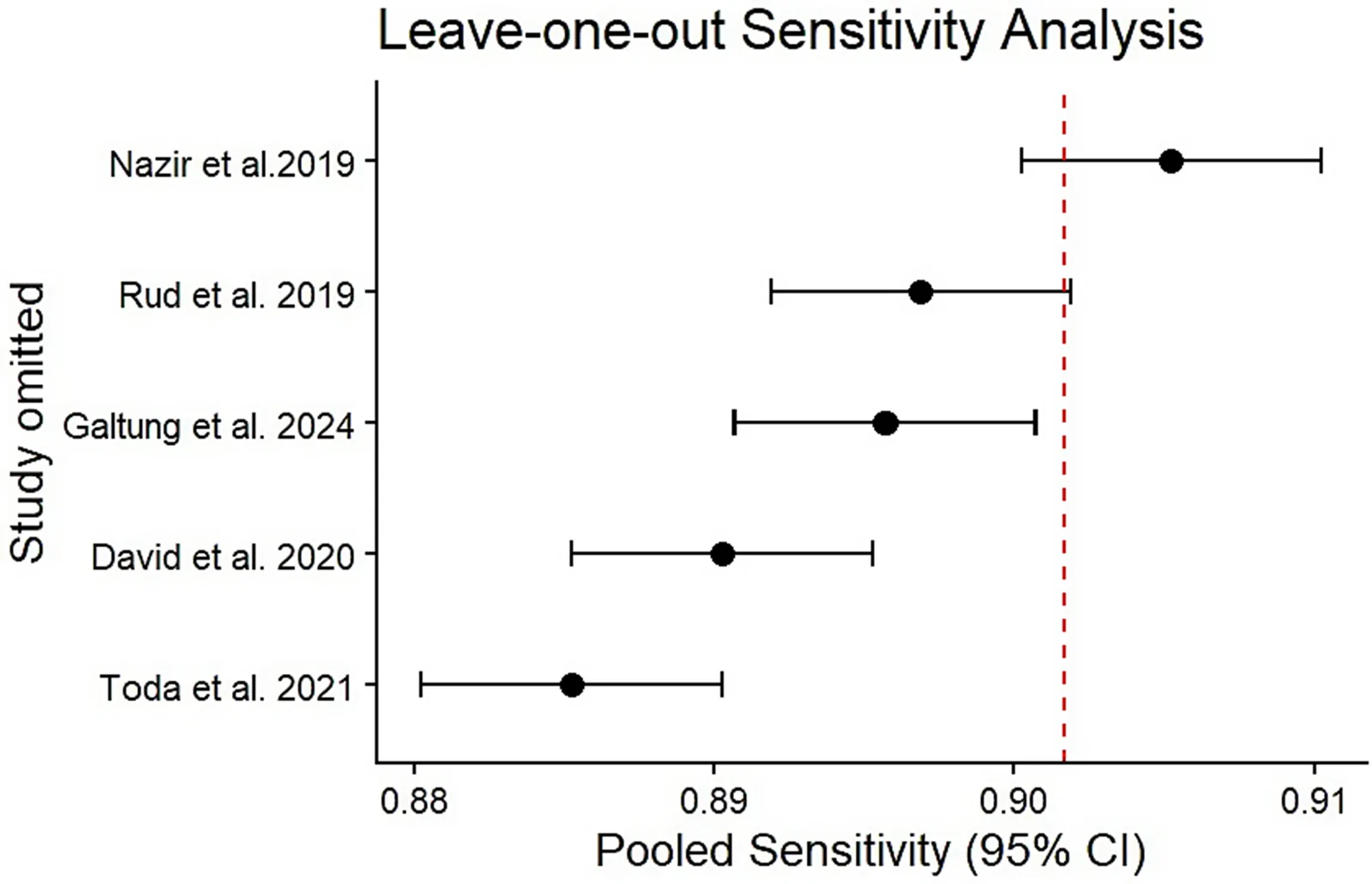
Leave-one-out sensitivity analysis for the diagnostic performance of CT urography in the detection of renal cell carcinoma (RCC)

Sensitivity analysis demonstrated the robustness of the pooled diagnostic accuracy estimates for renal cell carcinoma. Restricting the meta-analysis to prospective studies yielded a pooled sensitivity of 0.88 (95% CI: 0.65–0.96), a pooled specificity of 0.94 (95% CI: 0.81–0.98), and an AUC of 0.963, with low heterogeneity (I^2^ = 0%). Restricting the analysis to studies with a low risk of bias produced a pooled sensitivity of 0.94 (95% CI: 0.75–0.98), a pooled specificity of 0.97 (95% CI: 0.95–0.98), and an AUC of 0.976, with heterogeneity of 28.3%. Leave-one-out sensitivity analysis demonstrated that sequential exclusion of individual studies resulted in only minimal variation in pooled sensitivity (0.886–0.905) relative to the original pooled estimate (0.90), confirming that the overall findings were stable and were not unduly influenced by any single study (Fig. 6).

### Test quality for the detection of benign / non-malignant lesions

Only one eligible study evaluated the diagnostic performance of CTU for Benign / Non-Malignant conditions. The study demonstrated high diagnostic performance, with sensitivities ranging from 80% to 87% and specificities ranging from 95% to 98% across the evaluated benign urinary tract conditions.

### Phase-wise diagnostic accuracy of CT urography for UC

A phase-wise subgroup meta-analysis compared the diagnostic performance of single-, tri-, and four-phase CTU for detecting Urothelial carcinoma. The analysis included 12 single-phase, 12 tri-phase, and 10 four-phase CTU datasets.

**Fig. 7:**
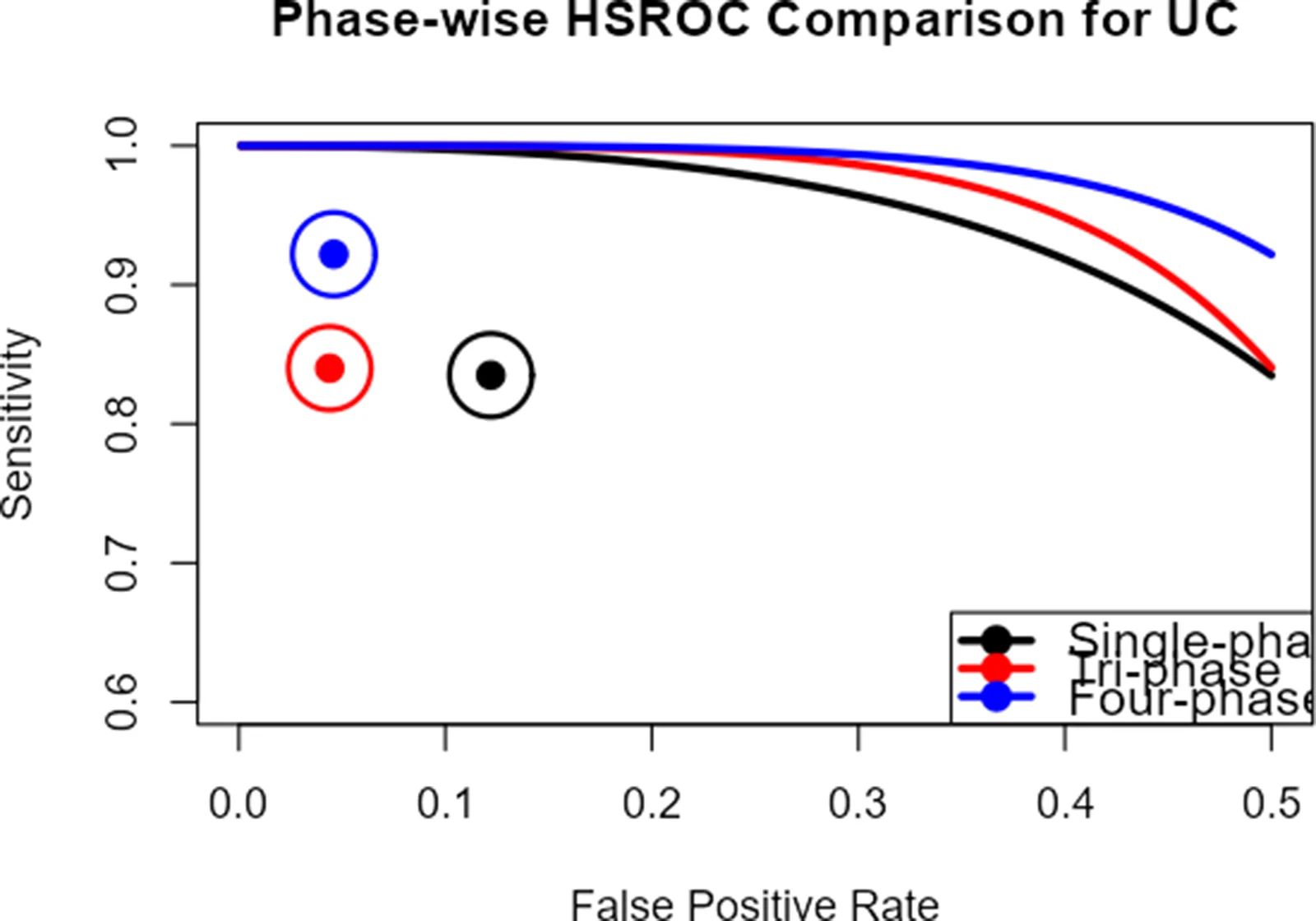
Comparison of HSROC curves for single-phase, tri-phase, and four-phase CTU for the detection of Urothelial carcinoma. The AUCs were 0.91,0.94, and 0.927, respectively. The circles represent the pooled summary estimates with 95% confidence regions, and the curves represent the corresponding HSROC models

The single-phase CTU demonstrated a pooled sensitivity of 0.835 (95% CI: 0.77–0.88) and specificity of 0.87 (95% CI: 0.82–0.91), with an AUC of 0.91. The tri-phase CTU demonstrated a similar sensitivity of 0.84 (95% CI: 0.77–0.88), but a markedly higher specificity of 0.95 (95% CI: 0.92–0.97), with an AUC of 0.94. Four–phase CTU achieved the highest sensitivity of 0.92 (95% CI: 0.89–0.94) and high specificity of 0.95 (95% CI: 0.87–0.98) with an AUC of 0.927. The HSROC (Fig. 7) comparison indicated that multiphasic protocols, especially tri- and four-phase CTU, offered superior overall diagnostic accuracy compared with single-phase CTU. Sensitivity analysis excluding studies with non-standard CTU protocols (portal venous and Bladder scan phases) yielded comparable pooled estimates.

### Phase-wise diagnostic accuracy of CT urography for RCC

A phase-wise meta-analysis for RCC could not be performed because only two studies were available in each phase group, and several contained Zero-cell values that prevented model convergence. Consequently, phase-specific pooled estimates could not be generated. Based on narrative synthesis, CTU demonstrated consistently high diagnostic accuracy across phases. The single-phase CTU showed a sensitivity of 84.8–100% and a specificity of 90.2–98%. Tri-phase CTU reported sensitivity of 70.2–100% and specificity of 81.4–100%, with some perfect values attributable to zero-cell counts. Four-phase CTU demonstrated uniformly high sensitivity (100%) and specificity (97–100%). Although performance appeared similar across phases, the limited number of studies restricts meaningful statistical comparison.

### Phase-wise diagnostic accuracy of CT urography for benign/ non-malignant lesions

A phase-wise meta-analysis for benign or non-malignant lesions was not feasible because only one eligible study evaluated these conditions. Descriptive analysis demonstrated comparable diagnostic performance between single-phase and tri-phase CTU protocols. Single-phase CTU demonstrated a sensitivity of 80% and specificity ranging from 96 to 98%, whereas tri-phase CTU demonstrated similarly high accuracy with sensitivity of 84–87% and specificity of 95–98%. Overall, both protocols performed comparably, with consistently high diagnostic accuracy, although limited study numbers prevented formal statistical comparison.

### Radiation dose in tri-phase CT urography

Effective dose (ED) was reported in 10 of the included studies (Table 2). Reported ED values ranged from 1.1 mSv for a low-dose phase-specific acquisition to 35.04$\:\pm\:$16.8 mSv for a conventional multiphasic CTU examination. Among studies comparing optimized low-dose and conventional CT urography protocols, lower effective doses were observed with the optimized protocols. Low-dose protocols generally reported ED values ranging from 2.01$\:\pm\:$0.44 mSv to 13.0$\:\pm\:$3.1 mSv, whereas conventional protocols ranged from 5.7 mSv to 22.5 mSv, with one study reporting an ED of 35.04$\:\pm\:$16.8 mSv.

### Meta-analytic estimate of CTDI_vol_

**Fig. 8:**
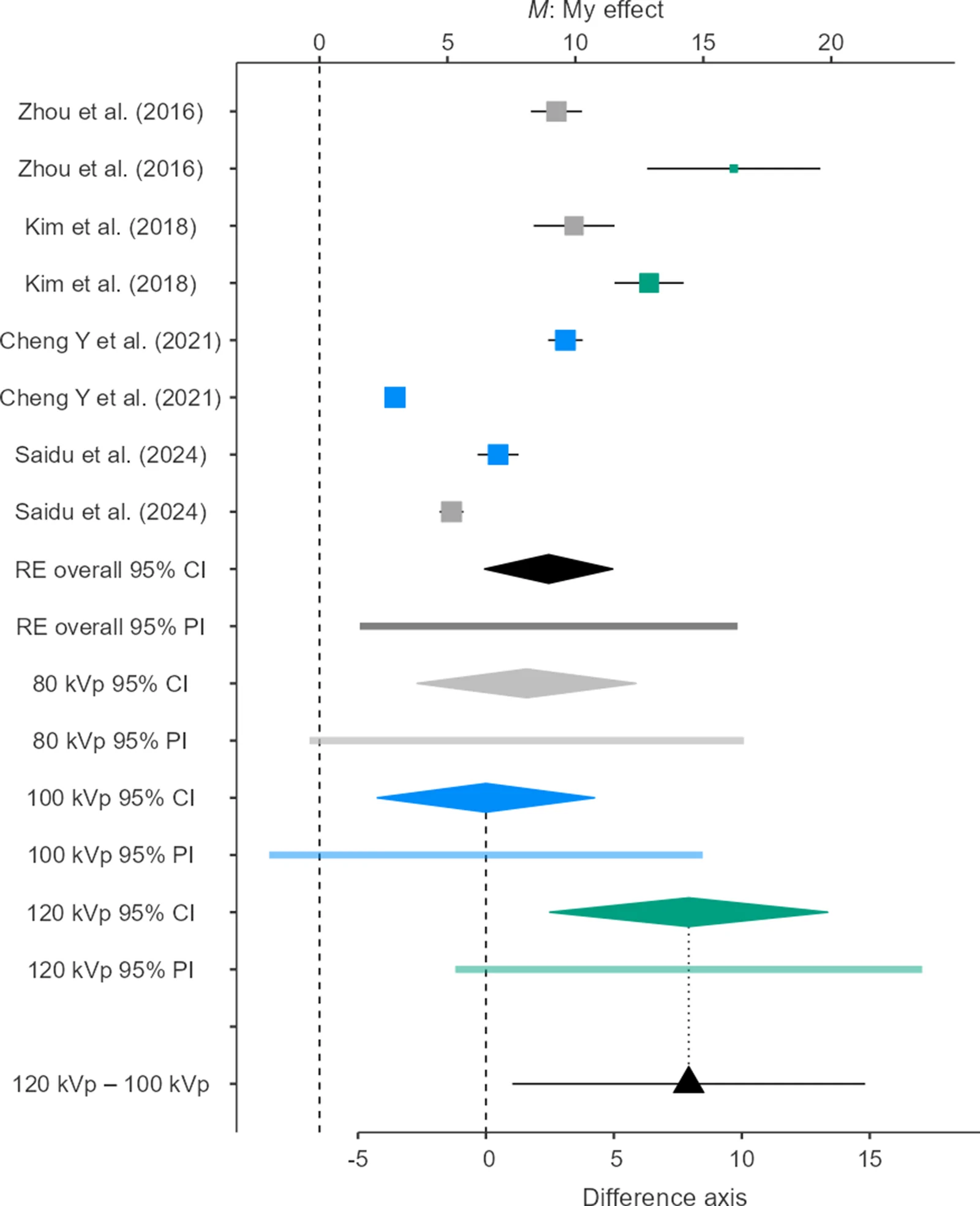
Forest plot showing the pooled mean CTDI_vol_ estimates by tube voltage using a random-effect meta-analysis. Diamonds represent 95% confidence intervals (CI), and horizontal bars represent 95% prediction intervals (PI)

**Fig. 9:**
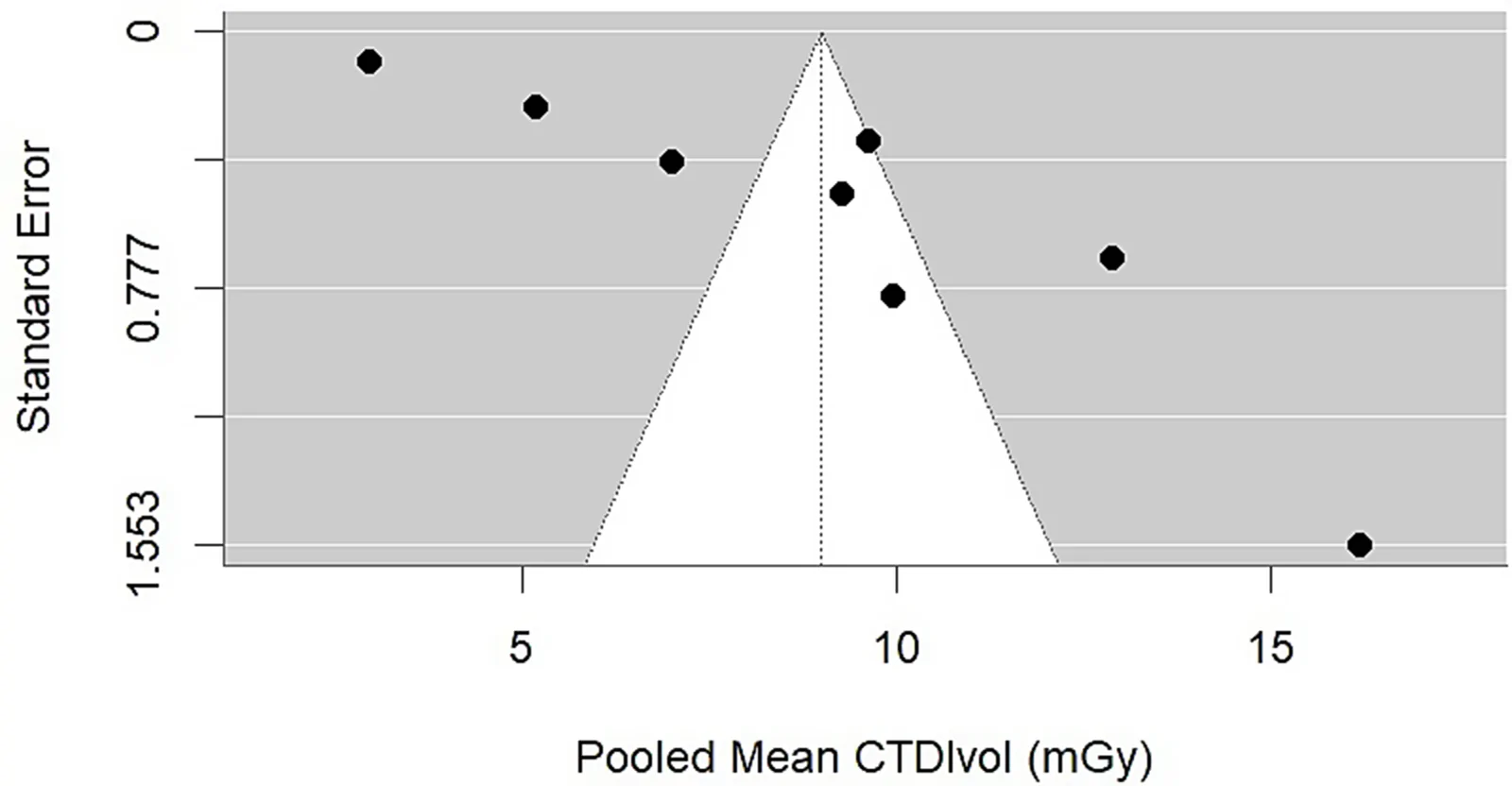
Funnel plot assessing potential publication bias in the CTDI _vol_ meta-analysis

A random-effects meta-analysis of eight CTU protocols yielded a pooled mean CTDI_vol_ of 8.96 mGy (95% CI: 6.46–11.5), with a 95% prediction interval of 1.57–16.3 mGy and a Diamond ratio of 17.01 (Fig. 8). Considerable heterogeneity between studies was observed (I^2^ = 99.24%). Subgroup analysis was performed according to tube voltage. The pooled mean CTDI_vol_ was 14.43 mGy (95% CI: 9–19.9) for 120 kVp protocols, 8.09 mGy (95% CI: 3.82–12.4) for 80 kVp protocols, and 6.5 mGy (95% CI: 2.26–10.7) for 100 kVp protocols. Moderator analysis demonstrated a significantly higher pooled CTDI_vol_ for 120 kVp compared with 100 kVp protocols, with a mean difference of 7.93 mGy (95% CI: 1.03–14.8). Despite subgroup analysis, substantial heterogeneity persisted within the 80 kVp (I^2^ = 97.6%), 100 kVp (I^2^ = 99.6%), and 120 kVp (I^2^ – 73.7%) subgroups. A funnel plot was generated to assess potential publication bias. Visual inspection suggested slight asymmetry (Fig. 9).

### Sensitivity analysis

Sensitivity analysis demonstrated the robustness of the pooled CTDI _vol_ estimates. Restricting the meta-analysis to prospective studies with low risk of bias yielded a pooled mean CTDI_vol_ of 7.87 mGy (95% CI: 5.16–10.6), with substantial heterogeneity (I^2^ = 99.33%). Compared with primary analysis (8.96 mGy), the pooled estimate showed only a modest reduction, indicating that exclusion of retrospective studies with some concerns did not materially alter the overall findings. Leave-one-out sensitivity analysis further demonstrated that sequential exclusion of individual protocol arms resulted in pooled CTDI_vol_ estimates ranging from 8.07 to 9.84 mGy, confirming that the overall findings were stable and were not disproportionately influenced by any single study.

### Meta-analytic estimate of the DLP

**Fig. 10:**
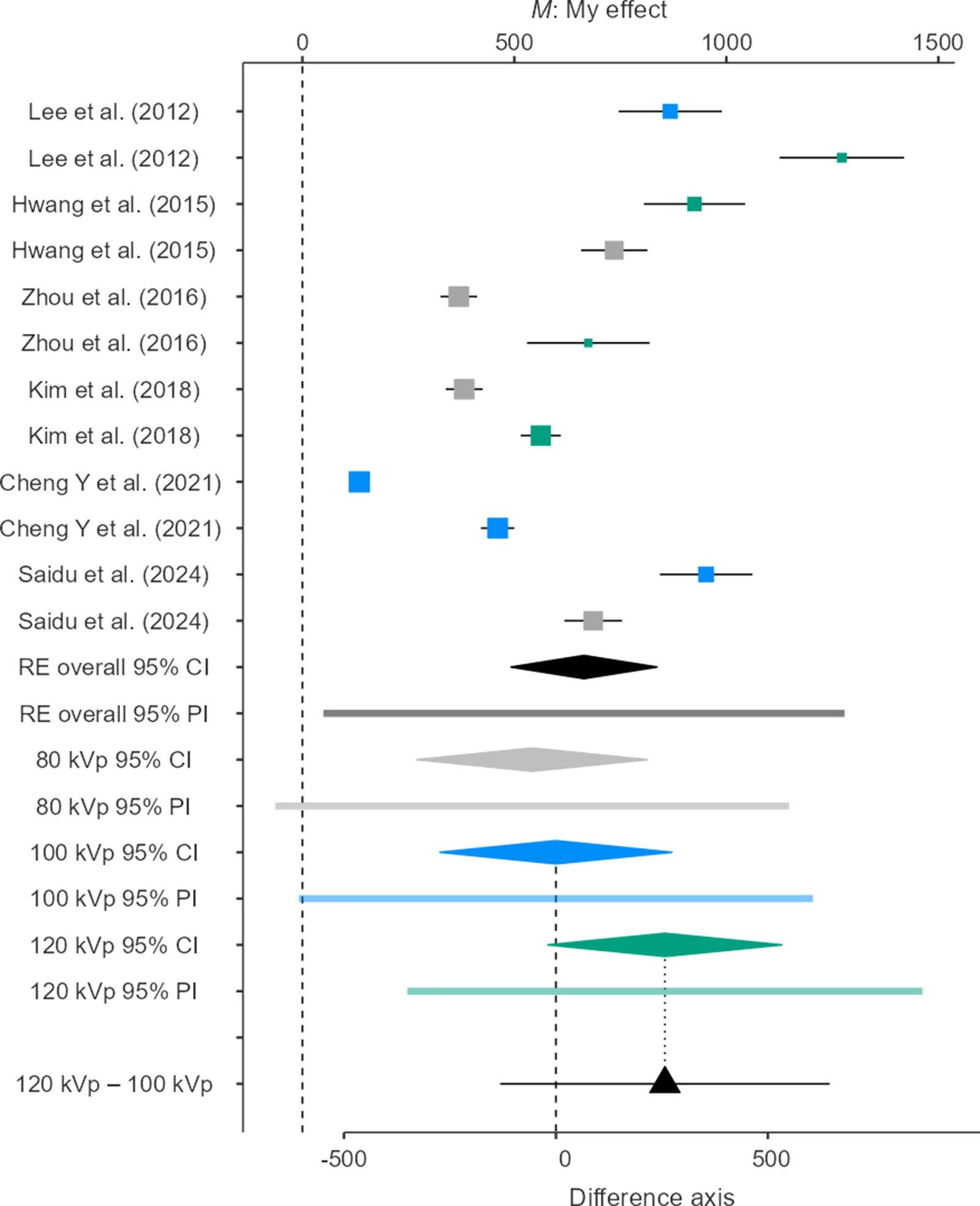
Forest plot showing the pooled mean DLP estimates by tube voltage using a random-effect meta-analysis. Diamonds represent 95% confidence intervals (CI), and horizontal bars represent 95% prediction intervals (PI)

**Fig. 11:**
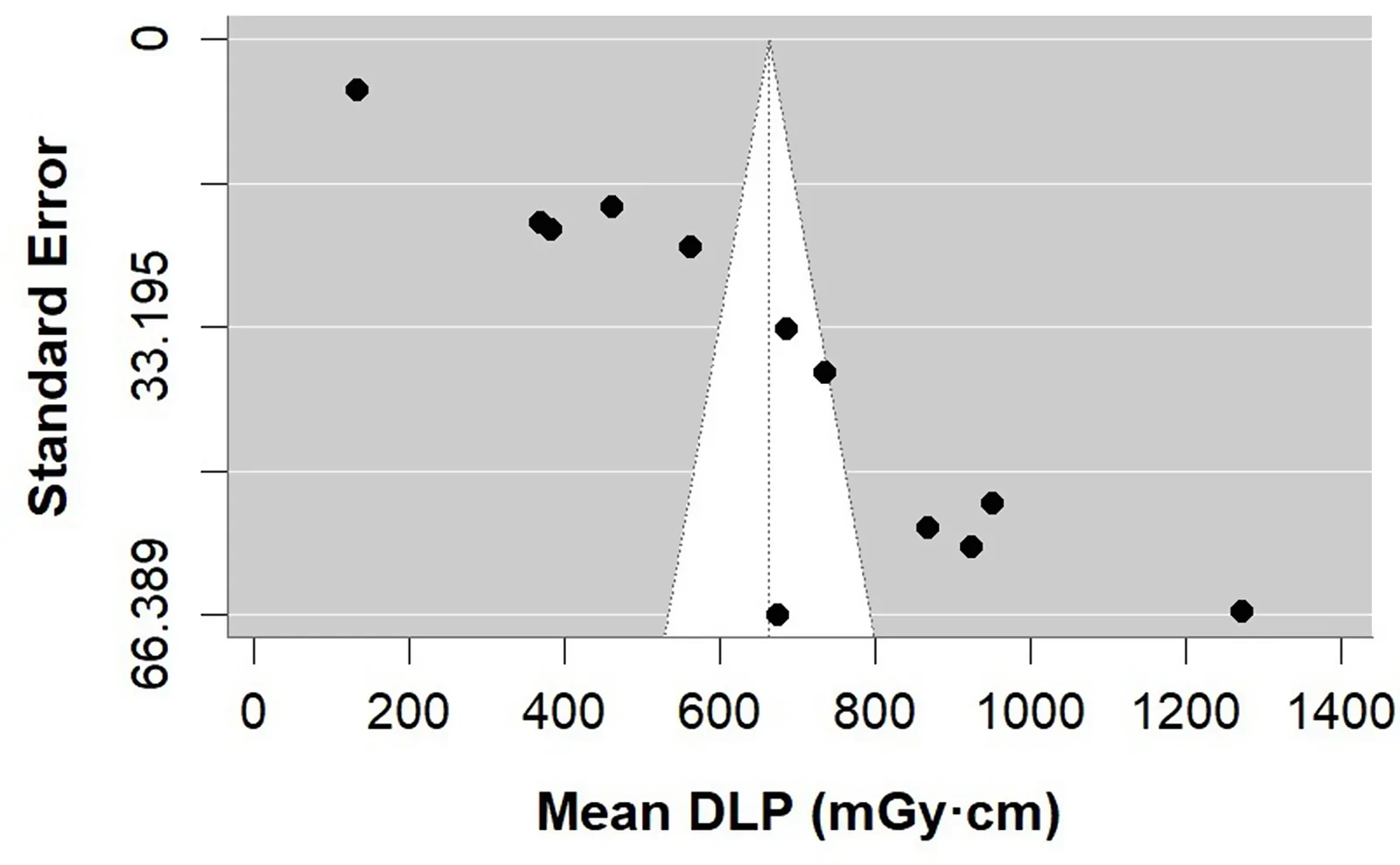
Funnel plot for assessment of potential publication bias in the DLP meta-analysis

A random-effects meta-analysis of 12 CT urography protocols yielded a pooled means DLP of 664 mGy.cm (95% CI: 492–836), with a 95% prediction interval of 49.53–1279 mGy.cm and a Diamond ratio of 17.94 (Fig. 10). Considerable between-study heterogeneity was observed (I2 = 99.39%). Subgroup analysis by tube voltage was performed. The pooled mean DLP was 855 mGy.cm (95% CI: 579–1131) for 120 kVp protocols, 598 mGy.cm (95% CI: 325–871) for 100 kVp protocols, and 542 mGy.cm (95% CI: 270–814) for 80 kVp protocols. Although the pooled DLP was numerically higher for 120 kVp than for 100 kVp protocols, moderator analysis showed that this difference was not statistically significant (mean difference: 257 mGy · cm, 95% CI: -131 to 646). High heterogeneity persisted within the 80 kVp (I^2^ = 97.67%), 100 kVp (I^2^ = 99.53%), and 120 kVp (I^2^ = 97.55%) subgroups. Potential publication bias was assessed using a funnel plot, which suggested slight visual asymmetry (Fig. 11).

### Sensitivity analysis

Restricting the analysis to prospective studies yielded a pooled mean DLP of 602 mGy.cm (95% CI: 394–809), with persistent high heterogeneity (I^2^ = 99.9%). Similarly, restricting the analysis to studies with a low risk of bias resulted in a pooled mean DLP of 694 mGy.cm (95% CI: 489–898), with similar high heterogeneity (I^2^ = 99.5%). Leave-one-out sensitivity analysis showed the sequential exclusion of individual protocol arms resulted in pooled mean DLP estimates ranging from 609.4 to 712.6 mGy.cm, with consistently high heterogeneity (I^2^ = 98.64–99.51%), indicating that no single protocol had a disproportionate influence on the overall pooled estimate.

### Risk of bias assessment

**Fig. 12:**
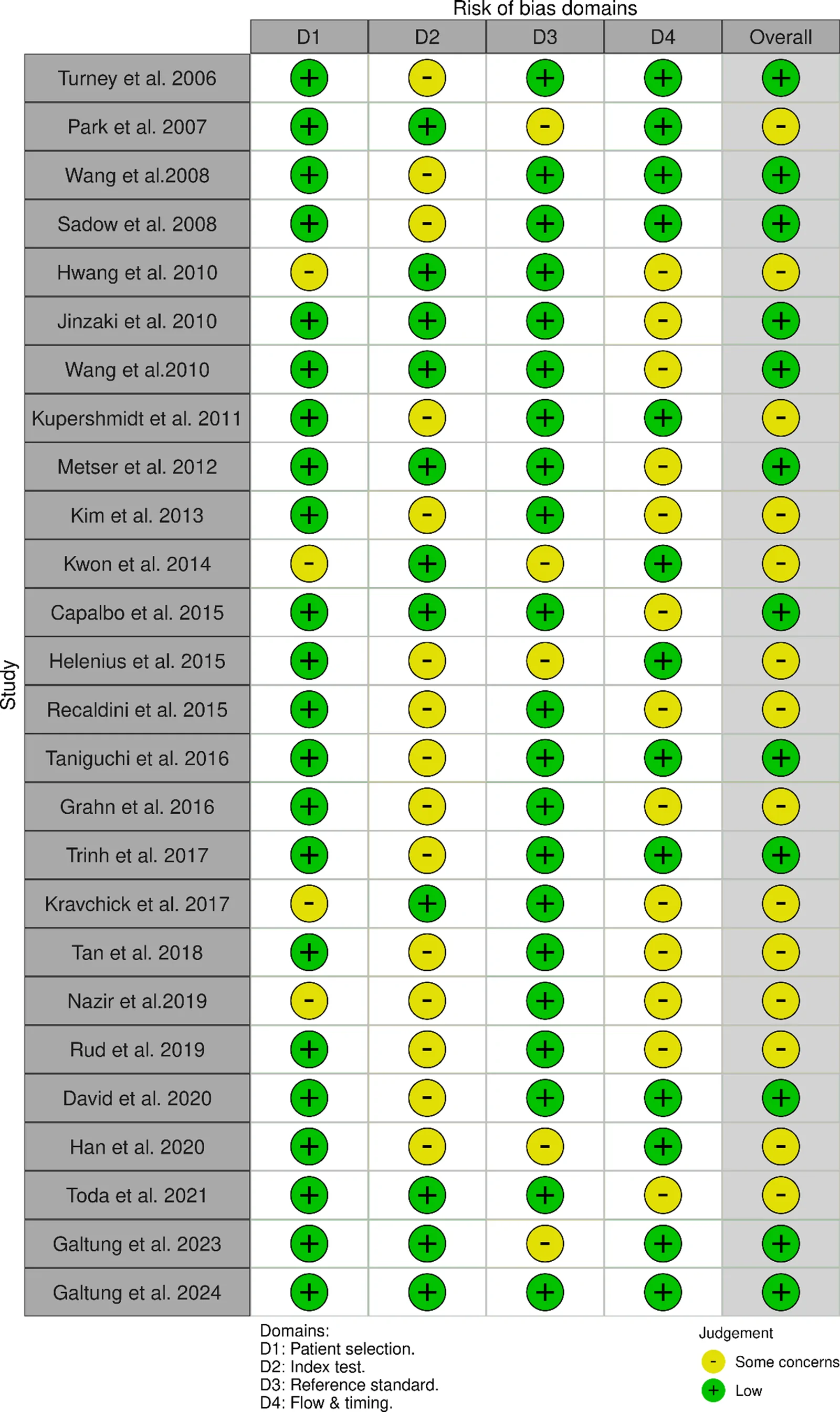
Risk of bias assessment for diagnostic accuracy studies using the QUADAS-2 tool across four domains: patient selection (D1), index test (D2), reference standard (D3), and flow and timing (D4). The green circles indicate a low risk of bias, and the yellow circles indicate some concerns

The methodological quality of the included studies was variable (Fig. 12; Table 3), reflecting differences between diagnostic accuracy investigations and radiation dose studies.

**Table 3:** Risk of bias assessment of radiation dose studies using the JBI critical appraisal checklist for analytical cross-sectional studies

Study	Q1: Criteria for Inclusion Clearly Defined	Q2: Description of Subjects and Setting	Q3: Valid and Reliable Exposure Measurement	Q4: Standard Outcome Measurement	Q5: Confounding Factors Identified	Q6: Strategies to Deal with Confounders	Q7: Valid and Reliable Outcome Measurement	Q8: Appropriate Statistical Analysis	Overall Risk of Bias
Meindl et al. (2007)	Yes	Yes	Yes	Yes	Yes	Yes	Yes	Yes	Low
Dahlman et al. (2011)	Yes	Yes	Yes	Yes	Yes	Yes	Yes	Yes	Low
Lee et al. (2012)	Yes	Yes	Yes	Yes	Yes	Yes	Yes	Yes	Low
Juri et al. (2013)	Yes	Yes	Yes	Yes	Yes	Yes	Yes	Yes	Low
Juri et al. (2013)	Yes	Yes	Yes	Yes	Yes	Yes	Yes	Yes	Low
Al-Amin et al. (2015)	Yes	Yes	Yes	Yes	Yes	Yes	Yes	Unclear	Low
Hwang et al. (2015)	Yes	Yes	Yes	Yes	Yes	Yes	Yes	Yes	Low
Zhou et al. (2016)	Yes	Yes	Yes	Yes	Unclear	No	Yes	Yes	Some concerns
Kim et al. (2018)	Yes	Yes	Yes	Yes	Yes	Yes	Yes	Yes	Low
Garba et al. (2019)	Yes	Yes	Yes	Yes	Unclear	No	Yes	Yes	Some concerns
Cheng Y et al. (2021)	Yes	Yes	Yes	Yes	Yes	Yes	Yes	Yes	Low
Saidu et al. (2024)	Yes	Yes	Yes	Yes	Yes	Yes	Yes	Yes	Low

Among diagnostic accuracy studies, patient selection was generally appropriate, with most studies enrolling populations that reflected routine clinical practice. The reference standards used—such as cystoscopy, ureteroscopy, and histopathology for urothelial malignancy—were suitable for the conditions under investigation. However, the type of reference standard differed across studies and disease categories, which limits direct comparison of diagnostic performance between CTU protocols and phase groups. In addition, several studies showed some concerns in the index test and flow-and-timing domains, most often because blinding procedures or the time interval between CTU and the reference standard were not clearly reported.

Radiation dose studies were, in general, methodologically sound, with most using standard dose metrics and appropriate measurement techniques. That said, a number of studies did not fully account for important confounding factors, including patient body habitus, scan length, and protocol individualization. These omissions likely contributed to the very high heterogeneity observed in pooled dose estimates (I² > 97%), which limits the interpretability of summary radiation dose values.

Overall, although most studies were judged to be of acceptable quality, variation in reference standards and incomplete control of confounders introduce uncertainty, particularly for pooled dose outcomes, and should be taken into account when interpreting the findings of this review.

### GRADE framework

According to the GRADE Framework, the certainty of evidence was high for urothelial carcinoma and moderate for renal cell carcinoma (Table 4a and b). Certainty assessment was not performed for benign/non-malignant conditions because only one eligible study was available.

**Table 4a:** GRADE framework: certainty of evidence for CT urography in the detection of urothelial carcinoma

Outcome	№ of studies (№ of patients)	Study design	Factors that may decrease certainty of evidence					Effect per 1,000 patients tested	Test accuracy CoE
			Risk of bias	Indirectness	Inconsistency	Imprecision	Publication bias	pre-test probability of 18%	
**True positives** (patients with Urothelial carcinoma)	20 studies 4418 patients	cross-sectional (cohort type accuracy study)	not serious	not serious	not serious	not serious	none	158 (153 to 164)	⨁⨁⨁⨁ High
**False negatives** (patients incorrectly classified as not having Urothelial carcinoma)								22 (16 to 27)	
**True negatives** (patients without Urothelial carcinoma)	20 studies 4418 patients	cross-sectional (cohort type accuracy study)	not serious	not serious	not serious	not serious	none	763 (738 to 787)	⨁⨁⨁⨁ High
**False positives** (patients incorrectly classified as having Urothelial carcinoma)								57 (33 to 82)	

Note- Sensitivity was 0.88 (95% CI: 0.85 to 0.91), and Specificity was 0.93 (95% CI: 0.90 to 0.96), Prevalence was 18%

**Table 4b:** GRADE framework: certainty of evidence for CT urography in the detection of renal cell carcinoma

Outcome	№ of studies (№ of patients)	Study design	Factors that may decrease certainty of evidence					Effect per 1,000 patients tested	Test accuracy CoE
			Risk of bias	Indirectness	Inconsistency	Imprecision	Publication bias	pre-test probability of 13%	
**True positives** (patients with Renal cell carcinoma (RCC))	5 studies 1496 patients	cross-sectional (cohort type accuracy study)	serious^a^	not serious	not serious	not serious	none	117 (103 to 124)	⨁⨁⨁◯ Moderate^a^
**False negatives** (patients incorrectly classified as not having Renal cell carcinoma (RCC))								13 (6 to 27)	
**True negatives** (patients without Renal cell carcinoma (RCC))	5 studies 1496 patients	cross-sectional (cohort type accuracy study)	serious^a^	not serious	not serious	not serious	none	844 (774 to 861)	⨁⨁⨁◯ Moderate^a^
**False positives** (patients incorrectly classified as having Renal cell carcinoma (RCC))								26 (9 to 96)	

Note- Sensitivity was 0.90 (95% CI: 0.79 to 0.95), and Specificity was 0.97 (95% CI: 0.89 to 0.99), Prevalence was 13%

a. Downgraded one level because 60% (3/5) of the included studies were rated as having some concerns on QUADAS-2. The concerns mainly related to patient selection, index test interpretation, and flow and timing, which may have introduced bias. Despite consistent pooled estimates in sensitivity analyses, a conservative one-level downgrade was applied

## Discussion

This review demonstrates that CTU achieves high diagnostic accuracy for urothelial and renal cell carcinoma across imaging protocols, with multiphasic examinations showing superior diagnostic performance compared with single-phase imaging. However, radiation dose reporting across studies was highly heterogeneous, limiting the interpretability of pooled dose estimates and highlighting the need for protocol optimization to balance diagnostic benefit with radiation safety.

### Diagnostic accuracy across pathologies

Across the studies reviewed, CTU demonstrated consistently strong diagnostic performance, although accuracy varied somewhat by condition. For urothelial carcinoma, pooled sensitivity and specificity (0.87 and 0.93) were similar to previously reported values, while results for renal cell carcinoma (0.90 and 0.97) showed a comparable pattern [68–70]. The AUC values of 0.94–0.97 further confirm its strong discriminatory ability. Accuracy for benign or non-malignant lesions was also high, with limited variation across studies. Overall, the evidence supports CTU as a dependable diagnostic tool for a wide range of urinary tract diseases.

### Influence of the acquisition phase on diagnostic accuracy

The phase-wise analysis showed that CTU performance varies notably with the imaging protocol. Single-phase CTU achieved acceptable accuracy, but its specificity was lower than that of multiphasic protocols [38, 44]. Tri-phase and four-phase CTU performed better for urothelial carcinoma, with specificity increasing from 0.88 in single-phase imaging to 0.96 and 0.95, respectively [32, 38, 52]. These improvements suggest that including the corticomedullary and excretory phases improves lesion characterization and reduces false-positive findings [42, 71, 72].

For renal cell carcinoma and benign conditions, the available evidence was limited, and the small number of eligible studies prevented a full phase-wise meta-analysis. Although narrative findings pointed to good accuracy across phases, the dataset was too small to support firm conclusions.

From a clinical perspective, these findings support an indication-driven approach to CTU protocol selection. Single-phase CTU may be appropriate in selecting low-risk or follow-up settings where radiation reduction is prioritized. Tri-phase CTU appears to offer a balanced compromise between diagnostic accuracy and radiation exposure for routine evaluation of suspected urothelial carcinoma, whereas four-phase CTU should be reserved for high-risk patients or diagnostically equivocal cases where maximal specificity or sensitivity is required.

### Radiation dose and dose-optimization strategies

Across the studies examining tri-phase CTU, the reported radiation doses showed substantial variation. The pooled mean CTDI_vol_ was roughly 10 mGy, but individual studies reported values ranging from about 5 mGy to almost 16 mGy. Comparable variability was seen in the DLP and effective-dose figures. Between study heterogeneity was extremely high (I² > 97%), indicating that pooled estimates should be interpreted as descriptive summaries rather than representative reference values. This level of heterogeneity does not undermine the findings but instead reflects the wide diversity of scanner platforms, scan coverage, acquisition parameters, phase numbers, and dose-modulation practices used across institutions.

The pooled mean DLP observed in the present review was lower than published European CTU dose benchmarks, including the Dutch national survey (median 708 mGy.cm; 75th percentile, 981.6 mGy.cm) [73], and recent Greek institutional DRL (75th percentile, 992–1252 mGy.cm) [74]. Similarly, an IAEA survey across 20 European-region countries demonstrated substantial variation in CTU DLP (740–3618 mGy.cm), reflecting differences in protocol design and the number of acquisition phases [9]. However, emphasis should be placed on protocol optimization to minimize radiation exposure while maintaining diagnostic performance.

Evidence from dose-reduction studies suggests that meaningful reductions can be achieved through relatively simple protocol adjustments. Lower tube-voltage settings (80–100 kVp), automatic tube-current modulation, and restricting the number of phases have each produced dose reductions of roughly 20–72% while maintaining diagnostic adequacy [10, 75–77]. Optimised low-dose protocols often report CTDI_vol_ values in the range of 2–9 mGy, substantially lower than those used in standard multiphasic CTU [78]. Although CTDI _vol_ is widely used to compare protocol performance, it does not account for patient size. Therefore, size-specific dose estimate (SSDE), recommended by the American Association of Physicists in Medicine (AAPM), provides a more patient-specific assessment of radiation dose by incorporating patient size into dose estimation. The combined use of CTDI_vol_ and SSDE can support a more individualized protocol optimization while maintaining adherence to the ALARA principle [79].

### Reconstruction-based dose optimization

Across the studies reviewed, reconstruction techniques had a noticeable impact on how much radiation could be reduced in CTU. Iterative reconstruction methods such as AIDR-3D, ASiR/ASiR-V, and SAFIRE generally improved noise performance and made it possible to use lower tube-current settings. Some studies reported that replacing filtered-back projection with AIDR-3D reduced CTDI_vol_ by more than half [59]. Similar reductions were seen with ASiR-based protocols, where adjustments in kVp or mAs produced meaningful dose savings while maintaining diagnostic usefulness [62, 63]. More recent work on deep-learning reconstruction shows even stronger potential, with better noise control and sharper detail than conventional iterative approaches, and these methods were linked with some of the lowest dose values in the dataset [65]. However, access to advanced reconstruction technologies is not uniform across institutions. Importantly, meaningful dose reduction remains achievable through widely available strategies such as phase reduction, low-kVp protocols in appropriate patients, and optimized automatic exposure control, indicating that effective CTU dose optimization does not depend solely on advanced reconstruction platforms.

### Balancing diagnostic benefit and radiation risk

Although additional CTU phases may enhance the detection of subtle lesions, the incremental diagnostic benefit is often limited in low-risk patients. Therefore, the decision to perform multiphasic CTU should be guided by an appropriate pre-test risk assessment. Previous recommendations have proposed stratifying patients according to age and the type of hematuria to identify those most likely to benefit from comprehensive CTU while minimizing unnecessary radiation exposure in lower-risk individuals [80, 81]. For patients classified as low risk, reduced-phase protocols are frequently sufficient to achieve adequate diagnostic confidence. Conversely, full multiphasic CTU should be reserved for patients with a higher clinical suspicion or equivocal findings, where the added diagnostic information justifies the increased radiation exposure. This approach is consistent with the ALARA principle, which advocates optimizing CTU protocols to achieve the required diagnostic information while minimizing unnecessary radiation exposure.

### Clinical implications

CTU remains an important tool for evaluating patients with gross haematuria or those with a high pre-test probability of urothelial carcinoma, consistent with current guideline recommendations. However, the results of this review suggest that multiphasic protocols should not be used routinely in all patients. Following appropriate pre-test risk assessment, patients with microscopic haematuria and no additional risk factors for urothelial malignancy may be adequately evaluated using reduced-phase CTU, thereby avoiding unnecessary radiation exposure.

In patients presenting with gross haematuria or a high pre-test probability of urothelial carcinoma, tri-phase CTU may provide a reasonable balance between diagnostic accuracy and radiation dose and can be considered for routine clinical practice. Four-phase CTU may be reserved for selected patients with persistent clinical suspicion of urothelial malignancy despite inconclusive initial imaging or equivocal findings requiring further characterization. Ultimately, protocol selection should be guided by the clinical indication rather than applying the same multiphasic approach to every patient.

### Limitations

This review has several limitations that should be acknowledged. Radiation dose outcomes showed very high heterogeneity (I² > 97%), which could only be partially explored through subgroup analysis based on tube voltage (kVp). Meta-regression and further exploration of heterogeneity were not feasible because important study- and patient-level covariates, including scanner generation, reconstruction method, scan coverage, dose modulation techniques, and patient body habitus, were not reported consistently or in a sufficient number of studies (typically at least 10 studies per covariate) to permit reliable analysis. In addition, effective dose estimates were derived using different DLP-to-effective dose conversion factors across the included studies, limiting direct comparability; therefore, effective dose values were summarized descriptively rather than quantitatively pooled.

Only English-language studies were included, which may have led to the exclusion of relevant work published in other languages. The study period covered nearly two decades, during which substantial advances occurred in CT hardware, dose modulation, and reconstruction techniques; however, temporal trends were not specifically examined. Important patient-related factors such as body mass index and body habitus were not consistently reported and could not be analyzed. Operator experience and institutional learning curves were also not addressed, and economic aspects, including the cost and availability of advanced reconstruction methods, were outside the scope of this review.

For diagnostic accuracy analyses, relatively few studies were available for renal cell carcinoma and benign conditions, limiting phase-wise comparisons. In particular, the small number of studies evaluating benign/non-malignant conditions precluded meaningful sensitivity analysis and formal certainty-of evidence assessment using the GRADE framework, thereby limiting the strength and certainty of the available evidence for this outcome. Formal assessment of publication bias was also not feasible for the RCC, benign/non-malignant, and ED analysis because the limited number of included studies precluded reliable evaluation. Several studies lacked detailed methodological reporting, and the exclusion of split-bolus and triple-bolus protocols may reduce the generalizability of the findings to centers that routinely use these techniques.

### Future recommendations

Future research in CTU should move beyond single-centre experiences toward well-designed, multicentre prospective studies that use clearly defined and reproducible protocols. In particular, there is a need for standardized reporting templates for CTU studies that consistently document acquisition phases, scan length, reconstruction methods, and radiation dose metrics (CTDI_vol_, DLP, and effective dose). Such standardization would greatly improve comparability across studies and strengthen future evidence synthesis.

The development of registry-based studies represents another important opportunity. Large, multicentre registries could enable ongoing monitoring of radiation dose trends, facilitate benchmarking across institutions, and help identify achievable dose ranges for different CTU protocols in routine clinical practice. Registry data would also allow evaluation of real-world practice patterns that are difficult to capture in controlled studies.

Further work is also needed to assess the cost-effectiveness of newer dose-reduction strategies, including advanced reconstruction techniques and emerging CT technologies. Understanding whether these approaches offer sufficient diagnostic and radiation-safety benefits relative to their cost is essential, particularly for centres with limited resources.

Finally, future studies should place greater emphasis on patient-centred outcomes, such as downstream testing, impact on clinical decision-making, patient anxiety, and cumulative radiation exposure over time. Incorporating these outcomes would help ensure that CTU protocol optimization is guided not only by technical performance but also by patient benefit and overall value of care.

## Conclusion

CT urography continues to play an important role in the assessment of urinary tract disease, particularly in patients with suspected urothelial carcinoma, with consistently high diagnostic performance observed across studies, despite substantial variation in radiation dose, suggesting that meaningful dose reduction is achievable through optimized CTU protocols without compromising diagnostic accuracy.

These findings support an indication-based approach to CTU, in which reduced-phase protocols are appropriate for patients with microscopic haematuria and a low pre-test probability of urothelial malignancy. In contrast, tri-phase CTU provides a reasonable balance between diagnostic performance and radiation exposure for many routine clinical scenarios involving patients with gross haematuria or an increased pre-test probability of urothelial carcinoma. More extensive multiphasic protocols should be reserved for patients with persistent clinical suspicion of malignancy or equivocal imaging findings. However, because radiation dose estimates demonstrated substantial heterogeneity (I^2^ > 97%), the pooled dose values should be interpreted as descriptive summaries rather than clinical dose benchmarks. Continued implementation of optimization strategies, including phase reduction, appropriate tube voltage selection, exposure control, and patient size-based dose assessment using SSDE, is essential to achieve the ALARA principle while maintaining diagnostic image quality.

## Supplementary Information

Below is the link to the electronic supplementary material.

Supplementary Material 1

## Data Availability

All data generated or analysed during this study are included in this published article and its supplementary information files.

## Abbreviations

CT: Computed Tomography
CTU: Computed Tomography Urography
CTDI _vol_: Computed tomography Dose Index Volume
DLP: Dose Length Product
DRLs: Diagnostic Reference Levels
ED: Effective Dose
SSDE: Size–Specific Dose estimate
ESUR: European Society of Urogenital Radiology
GRADE: Grading of Recommendations Assessment, Development and Evaluation
HSROC: Hierarchical summary Receiver Operating Characteristic
IAEA: International Atomic Energy Agency
ICRP: International Commission on Radiological Protection
JBI: Joanna Briggs Institute
QUADAS: 2–Quality Assessment of Diagnostic Accuracy Studies 2
