# Supplementary material for: Diagnostic accuracy of single-, tri-, and four-phasic CT urography and radiation dose assessment: a systematic review and meta-analysis
Source: BMC Med Imaging. 2026 Aug 18;26:611. doi: 10.1186/s12880-026-02690-9 (PMC13757198; doi:10.1186/s12880-026-02690-9)
Supplement: Supplementary file 1 [file 12880_2026_2690_MOESM1_ESM.docx]

**Table S1.** Mapping of CT urography acquisition phases and study eligibility for the primary and phase-wise urothelial carcinoma meta-analyses.

| **SL. No** | **Study** | **CTU acquisition phases** | **Assigned phase category** | **TP** | **FP** | **FN** | **TN** | **Eligibility for quantitative synthesis** |
| --- | --- | --- | --- | --- | --- | --- | --- | --- |
| 1 | Turney et al. 2006 | UE, CM1,CM2 | Tri-phase | 42 | 1 | 3 | 115 | Included in both Main and Phasewise |
| 2 | Park et al. 2007 | UE, CMP, PP, EP | Four-phase | 60 | 4 | 3 | 51 | Included in both Main and Phasewise |
| 3 | Wang et al.2008 | UE, AP, NP, EP | Four-phase | 74 | 6 | 3 | 483 | Included in both Main and Phasewise |
| 4 | Sadow et al. 2008 | UE, NP, EP | Tri-phase | 117 | 40 | 32 | 649 | Included in both Main and Phasewise |
| 5 | Hwang et al. 2010 | UE, NP, EP | Tri-phase | 26 | 2 | 4 | 61 | Included in both Main and Phasewise |
|  |  | UE, NP, EP | Tri-phase | 15 | 5 | 10 | 20 |  |
| 6 | Jinzaki et al. 2010 | UE, NP, EP | Tri-phase | 43 | 3 | 3 | 55 | Included in both Main and Phasewise |
|  |  | EP | Single-phase | 37 | 11 | 9 | 47 | Included only in Phasewise |
| 7 | Wang et al.2010 | UE, AP, NP, EP | Four-phase | 23 | 0 | 1 | 215 | Included in both Main and Phasewise |
| 8 | Kupershmidt et al. 2011 | PV | Single-phase | 20 | 1 | 0 | 39 | Included in both Main and Phasewise |
| 9 | Metser et al. 2012 | UP, EP | Dual-phase | 30 | 12 | 4 | 114 | Included only in Main |
|  |  | UP, EP | Dual-phase | 59 | 16 | 5 | 80 | Included only in Main |
|  |  | UP | Single-phase | 29 | 5 | 5 | 121 | Included only in Phase-wise |
|  |  | EP | Single-phase | 27 | 10 | 7 | 116 | Included only in Phase-wise |
|  |  | UP | Single-phase | 58 | 10 | 6 | 86 | Included only in Phase-wise |
|  |  | EP | Single-phase | 47 | 8 | 17 | 88 | Included only in Phase-wise |
| 10 | Kim et al. 2013 | NP | Single-phase | 56 | 18 | 6 | 200 | Included only in Phase-wise |
|  |  | Pyelographic phase | Single-phase | 45 | 44 | 17 | 174 | Included only in Phase-wise |
|  |  | NP | Single-phase | 20 | 48 | 2 | 210 | Included only in Phase-wise |
|  |  | Pyelographic phase | Single-phase | 16 | 87 | 6 | 171 | Included only in Phase-wise |
|  |  | NP, Pyelographic phase | Dual-phase | 57 | 38 | 5 | 180 | Included only in Main |
|  |  | NP, Pyelographic phase | Dual-phase | 18 | 24 | 4 | 234 | Included only in Main |
| 11 | Capalbo et al. 2015 | UE, AP, NP, EP | Four-phase | 106 | 4 | 8 | 51 | Included in both Main and Phasewise |
| 12 | Helenius et al. 2015 | UE, CMP, NP, EP | Four-phase | 48 | 5 | 7 | 375 | Included in both Main and Phasewise |
| 13 | Recaldini et al, .2015 | UE, NP, EP | Tri-phase | 19 | 7 | 2 | 38 | Included in both Main and Phasewise |
| 14 | Grahn et al. 2016 | UE, AP, NP, EP | Four-phase | 55 | 20 | 7 | 21 | Included in both Main and Phasewise |
| 15 | Trinh et al. 2017 | UE, NP, EP | Tri-phase | 32 | 39 | 5 | 486 | Included in both Main and Phasewise |
|  |  | UE, NP, EP | Tri-phase | 50 | 8 | 8 | 82 | Included in both Main and Phasewise |
| 16 | Kravchick et al. 2017 | UE, NP, EP | Tri-phase | 6 | 2 | 3 | 129 | Included in both Main and Phasewise |
|  |  | UE, NP, EP | Tri-phase | 13 | 3 | 4 | 120 | Included only in Phase-wise |
| 17 | Tan et al. 2018 | NR | NR | - | - | - | - | Not included |
|  |  | NR | NR | - | - | - | - | Not included |
| 18 | Rud et al. 2019 | UE, CMP, NP,EP | Four-phase | 7 | 1 | 0 | 368 | Included in both Main and Phasewise |
| 19 | David et al. 2020 | UE,NP,EP | Tri-phase | 12 | 5 | 0 | 558 | Included only in Phase-wise |
|  |  | UE,NP,EP | Tri-phase | 4 | 5 | 0 | 566 | Included in both Main and Phasewise |
| 20 | Han et al. 2020 | UE, BS,NP,EP | Four-phase | 92 | 4 | 6 | 90 | Included in both Main and Phasewise |
| 21 | Galtung et al. 2023 | UE, CMP, NP, EP | Four-phase | 40 | 28 | 2 | 192 | Included in both Main and Phasewise |
|  |  | NP | Single-phase | 42 | 23 | 4 | 215 | Included only in Phase-wise |
|  |  | UE, CMP, NP, EP | Four-phase | 36 | 25 | 3 | 202 | Included in both Main and Phasewise |
|  |  | NP | Single-phase | 38 | 22 | 2 | 214 | Included only in Phase-wise |

CT urography protocols were classified according to the number and type of acquisition phases reported in the original studies (single-, dual-, tri-, or four-phase). Studies evaluating multiple protocols contributed separate datasets according to the reported phase combinations. Studies lacking sufficient 2 × 2 contingency data were excluded from the quantitative meta-analysis.
